# Oxidation-Triggered Formation of Diradical Cations from Paramagnetic Molecules and Their Spin Density Evolution

**DOI:** 10.3390/molecules30091931

**Published:** 2025-04-26

**Authors:** Di Wang, Dan Yao, Xinyu Li, Lingli Shi, Chunyuan Wang, Jie Li, Weili Kong, Yongliang Qin, Martin Baumgarten

**Affiliations:** 1 School of Materials Science and Chemical Engineering, Anhui Jianzhu University, Hefei 230601, China; 19719270397@163.com (D.Y.);; 2 Anhui Province Key Laboratory of Condensed Matter Physics at Extreme Conditions, Hefei Institutes of Physical Science, Chinese Academy of Sciences, Hefei 230031, China; 3 Max Planck Institute for Polymer Research, 55128 Mainz, Germany; baumgart@mpip-mainz.mpg.de

**Keywords:** diradical cations, spin characteristics and magnetic properties, controllable and reversible

## Abstract

Controllable intramolecular spin-polarized flow refers to the manipulation of spin-polarized electron transport within molecules through externally applied stimuli, thereby modulating their intramolecular spin characteristics and magnetic properties. In this work, we designed and synthesized four paramagnetic molecules, PDTN-NN, PDTN-IN, PO-NN, and PO-IN, by introducing nitronyl nitroxide (NN) and iminonitroxide (IN) radicals into phenothiazine and phenoxazine frameworks. Remarkably, we successfully generated the corresponding radical-substituted radical cations (diradical cations) and controlled their spin density distributions (SDDs) through redox stimuli. UV-Vis absorption spectroscopy, cyclic voltammetry (CV), electron paramagnetic resonance (EPR), and density functional theory (DFT) were employed to confirm the formation of diradical cations during the redox processes. Furthermore, EPR spectroscopy and DFT calculations were also employed to provide clear evidence of intramolecular magnetic coupling in the diradical cations.

## 1. Introduction

Stable open-shell molecules with redox activity have attracted considerable attention in chemistry and materials science, particularly in the synthesis and characterization of high-spin cation radicals [1,2,3]. Nitronyl nitroxide (NN) and imino nitroxide (IN) [4,5] are particularly appealing due to their high kinetic stability and structural versatility. They have become important spin-functional groups in organic synthesis [6,7,8,9,10]. Phenothiazine and phenoxazine have been widely recognized as classical electron donors over the past two to three decades [11,12,13,14,15]. Their distinctive planar structures with a ten-atom π-system containing a secondary amine are redox-active and can be converted into cation radicals upon electron loss [16,17]. However, classical cation radicals such as triphenylamine, synthesized by S. Suzuki and co-workers, display high spin density but are highly unstable, often undergoing para-coupling to form benzidine. To enhance stability, incorporating oxygen and sulfur atoms can modulate electron distribution [1,2,3]. K. Okada and coworkers developed a series of phenothiazine-based cation radicals which are chemically stable and nearly planar [18,19,20]. Further studies have focused on stabilizing cation radicals through free-radical modification. M. Kuratsu and coworkers introduced NN and IN radicals into azines and their derivatives, and through characterization they found that these compounds typically exhibit high-spin ground states. Theoretical calculations and magnetic studies have shown that such compounds have strong intramolecular interactions [2,3,21,22,23,24]. Their synthesis and research have broadened the way for obtaining high-spin molecular materials. It is worth noting that the regulation of intramolecular spin polarized electron transport through external stimuli enables effective manipulation of molecular spin characteristics and magnetic properties [25,26]. However, there is still a lack of systematic exploration and sufficient experimental evidence regarding the formation mechanisms of diradicals comprising both stable radicals and cation radicals including their fundamental physical and chemical processes and experimental investigations into the evolution of intramolecular spin characteristics and magnetic properties under varying redox conditions [27,28,29,30,31,32,33,34].

**Scheme 1:**
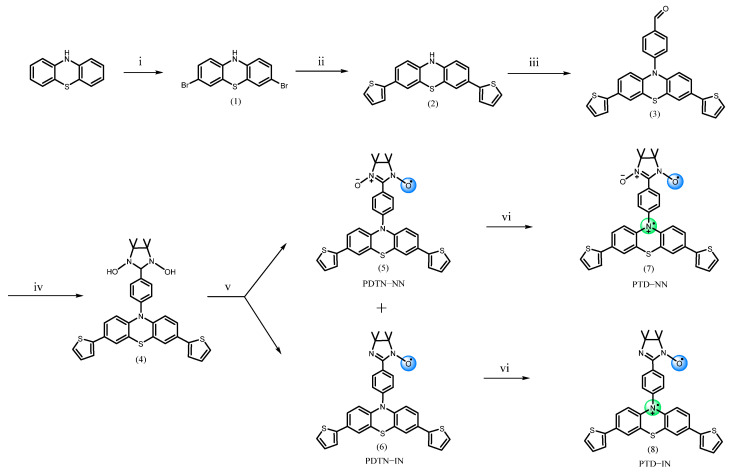
Synthesis of monoradicals PDTN-NN, PDTN-IN and diradical cations PTD-NN, PTD-IN. (i) NBS, THF, 0 °C, 1 h; (ii) thiophen-2-ylboronic acid, Pd(PPh_3_)_4_, K_2_CO_3_; (iii) 4-formylphenylboronic acid, TMEDA, Cu(NO_3_)_2_, CH_3_OH/THF; (iv) BHA, Toluene, 90 °C; (v) NaIO_4_, DCM/H_2_O; (vi) SbCl_5_. The blue and green circles represent distinct spin centers.

**Scheme 2:**
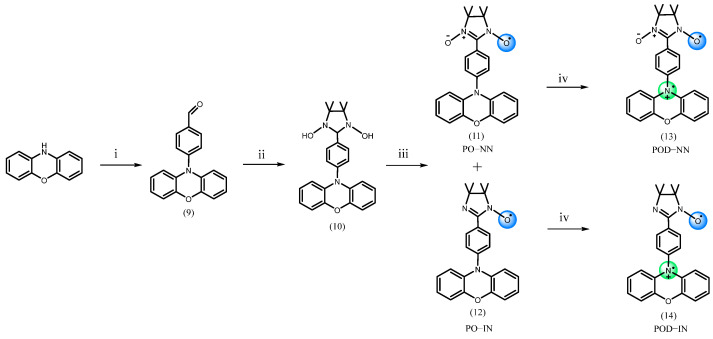
Synthesis of monoradicals PO-NN, PO-IN and diradical cations POD-NN, POD-IN. (i) 4-formylphenylboronic acid, TMEDA, Cu(NO_3_)_2_, CH_3_OH/THF; (ii) BHA, CH_3_OH, DCM; (iii) NaIO_4_, DCM/H_2_O; (iv) SbCl_5_. The blue and green circles represent distinct spin centers.

In this study, NN and IN radicals were functionalized with phenothiazine and phenoxazine. To modulate the electronic properties of the π-conjugated molecular framework, two thiophene groups were introduced at the 3,7-positions of phenothiazine, which resulted in a lower oxidation potential and facilitated the formation of cation radicals. We synthesized four new organic paramagnetic molecules; NN and IN radicals were connected with phenothiazine combined with two thiophenes at 3,7 positions and phenoxazine through 1,4-phenylene spacer [35], which were labeled PDTN-NN (5), PDTN-IN (6), PO-NN (11), and PO-IN (12), respectively (Scheme 1 and Scheme 2). Upon oxidation with SbCl_5_, we successfully obtained the corresponding diradical cations: PTD-NN (7), PTD-IN (8), POD-NN (13), and POD-IN (14). For comparison with these diradical cations, we synthesized mono-phenyl-substituted phenothiazine (PTP, 15) and its derivative PDTN (16), which carries two thiophene groups at the 3,7-positions. Their oxidation with SbCl_5_ led to the formation of the corresponding cation monoradicals, PTPC (17) and PDTC (18) (Scheme S1).

**Scheme 3:**
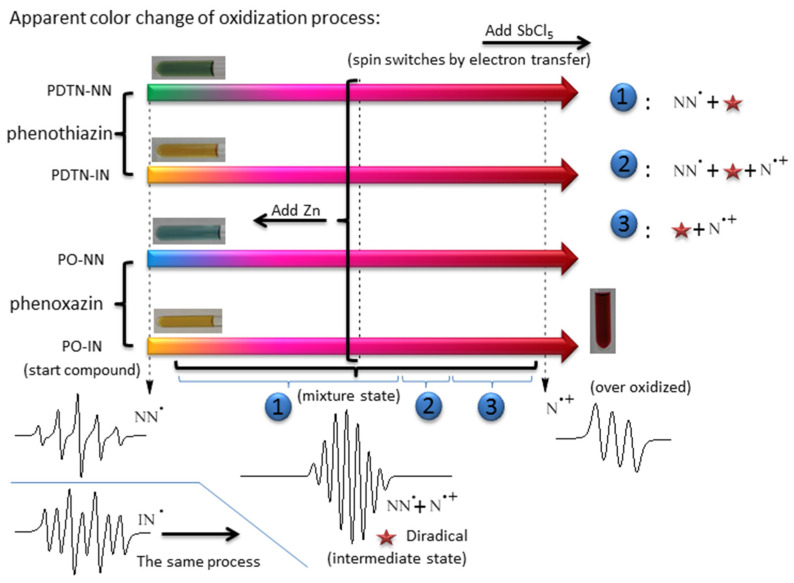
The apparent color and different oxidation stages of the four monoradicals in the redox process.

We performed oxidation experiments with SbCl_5_ for the four monoradicals in DCM [36,37]. The solution initially changed to pink, gradually deepened to deep pink, and finally turned red, as shown in Scheme 3. The mixture state can be divided into three stages, each with different components of radical species. Due to the oxidation of the secondary amine into nitrogen cation radical, the first mixture state consists of monoradicals and the newly formed diradical cations. Upon oxidation to the second mixture stage, a portion of the organic radicals was disrupted, leading to the formation of nitrogen cation radicals at this stage. By the third mixture stage, monoradicals no longer existed and the compound was composed of diradical cations and nitrogen cation monoradicals. The original quintet signal and heptet EPR signal patterns were affected by hyperfine splitting from nitrogen nuclei which belongs to the newly formed nitrogen cation radical (the nuclear spin angular momentum I = 1 for ^14^N), and the numbers of peaks incline to split more. Further oxidation pushed the system toward an over-oxidized state. At this stage, the EPR signal became increasingly similar to the typical nitrogen cation monoradical (three-line pattern spectrum). UV-Vis spectroscopy confirmed the formation of monoradicals and new radical species, and provided spectral evidence of structural evolution in nitroxide groups in response to increasing oxidant concentration. We utilized cyclic voltammetry to investigate electrochemical properties and oxidation processes of these four monoradicals, confirming the formation of diradicals. Using EPR, we investigated the spin switching of the four monoradicals at room temperature, observing hyperfine splitting influenced by the nitrogen cation radicals formed during oxidation [38,39]. With the reductant Zn, the EPR spectra reverted to the characteristic five- or seven-line patterns of monoradicals. By comparing experimental and simulated spectra, changes in the spin characteristics and magnetic properties of the radicals due to electron transfer were interpreted based on the pattern, number, position, and relative intensity of the EPR peaks. Additionally, DFT calculations were employed to analyze the spin density distributions in different redox states [40,41,42], and the intramolecular coupling constants of the diradical cations were also obtained through calculations.

## 2. Results

### 2.1. Optical Properties

#### 2.1.1. UV-Vis Absorption Spectra of Radicals with Phenothiazine and Phenoxazine

**Figure 1:**
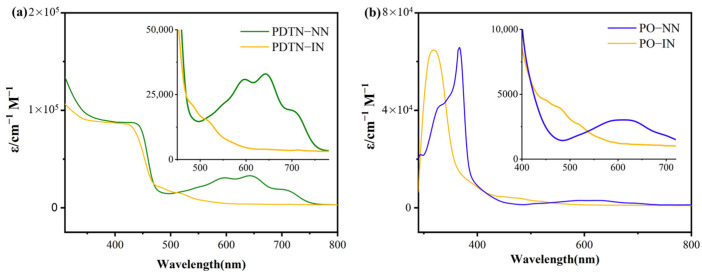
(**a**) UV-Vis absorption spectra of PDTN-NN and PDTN-IN recorded in DCM at room temperature (~10^−5^ M). Inset: amplification of optical absorption spectra in the visible range from 300 nm to 800 nm. (**b**) UV-Vis absorption spectra of PO-NN and PO-IN recorded in DCM at room temperature (~10^−5^ M). Inset: amplification of optical absorption spectra in the visible range from 390 nm to 800 nm.

The characteristic absorption spectra of NN and IN radicals functionalized with phenothiazine are shown in Figure 1a. The absorption peaks between 350 nm and 450 nm originate from the π-π* transition of the substituted dithiophene-phenothiazine-imidazolidine. For PDTN-NN, the main absorption of NN group originating from aminoxyl moiety ranges from 580 nm to 650 nm, and the maximum peak is 640 nm. For PDTN-IN, the characteristic absorption originating from aminoxyl moiety ranges from 470 nm to 520 nm, and the maximum absorption is around 480 nm. Therefore, this absorption region is the main characteristic absorption to distinguish NN and IN groups. Electron donor groups increase the energy level of the singly occupied molecular orbital (SOMO), thereby reducing the energy gap between the SOMO and the lowest unoccupied molecular orbital (LUMO) of the NN group. As a result, PDTN-NN appears green, whereas NN substituted with a phenyl group appears blue. The same principle was used to interpret UV-Vis absorption spectra of PO-NN and PO-IN as shown in Figure 1b. We can clearly distinguish the different absorptions of π-π* transition region which belong to phenoxazine and phenothiazine. The interactions of the two conjugated donor structures also have different effects on the nitroxide radicals which they connected to, especially to NN group. The characteristic absorptions of radicals to PO-NN are from 550 nm to 650 nm, and the maximum peaks are 590 nm and 625 nm. This also indicates that the electron donor ability of dithiophene-phenothiazine is stronger than phenoxazine group. The difference on structure leads the apparent color of PO-NN to light blue. The characteristic absorption of PO-IN, which also originates from aminoxyl moiety, is from 460 nm to 520 nm. The maximum absorption is around 480 nm.

#### 2.1.2. UV-Vis Absorption of Redox Stimuli Responsive Process

**Figure 2:**
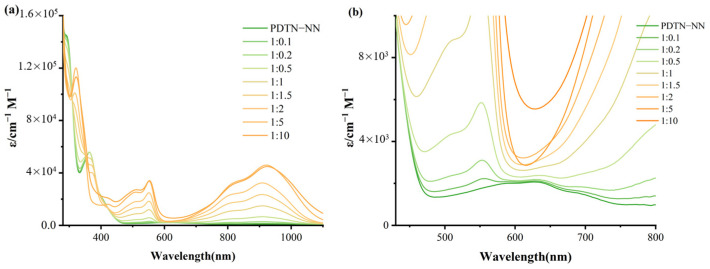
(**a**) UV-Vis absorption spectra of the whole oxidation titration process of PDTN-NN by adding different ratios of oxidants SbCl_5_ recorded in DCM at room temperature (~10^−5^ M). (**b**) Amplification of optical absorption spectra in the visible range from 430 nm to 800 nm.

We used UV-Vis absorption spectroscopy to monitor the oxidation titration of the four monoradicals with SbCl_5_ in DCM, employing a series of oxidant ratios from 0.1 to 10 equivalents. The substrate concentrations were maintained at 1.0 × 10^−5^ M to ensure the total concentration of solution remained constant during the whole redox titration process as shown in Figure 2 and Figures S2–S4, respectively. Figure 2 shows the spectrum change in the whole process of PDTN-NN oxidation. Two main absorption regions exhibited significant intensity enhancement: one is around 315 nm to 350 nm (the maximum absorption at 315 nm) and the other is around 500 nm to 580 nm (the maximum absorption at 555 nm) during the whole redox titration process. The significant changes in the spectrum provide evidence for the generation of diradical cations. Interestingly, we also observed an enhancement in the absorption peak in the near-infrared region around 800 to 1000 nm. This characteristic absorption of phenothiazine is likely due to the charge transfer between the lone pair electrons in the phenothiazine molecule and the entire conjugated system, resulting in near-infrared absorption. As oxidation progressed, the absorption bands associated with the imidazolidine and NN group completely disappeared when the SbCl_5_ ratio reached approximately five equivalents, indicating NN group was decomposed as the concentration of oxidation gradually increased. The general oxidation process and the critical points of structure transition of PDTN-IN, PO-NN and PO-IN are similar to PDTN-NN, as shown in Figures S2–S4. Only the peak positions of absorptions show slight differences.

### 2.2. Cyclic Voltammetry Measurements

**Figure 3:**
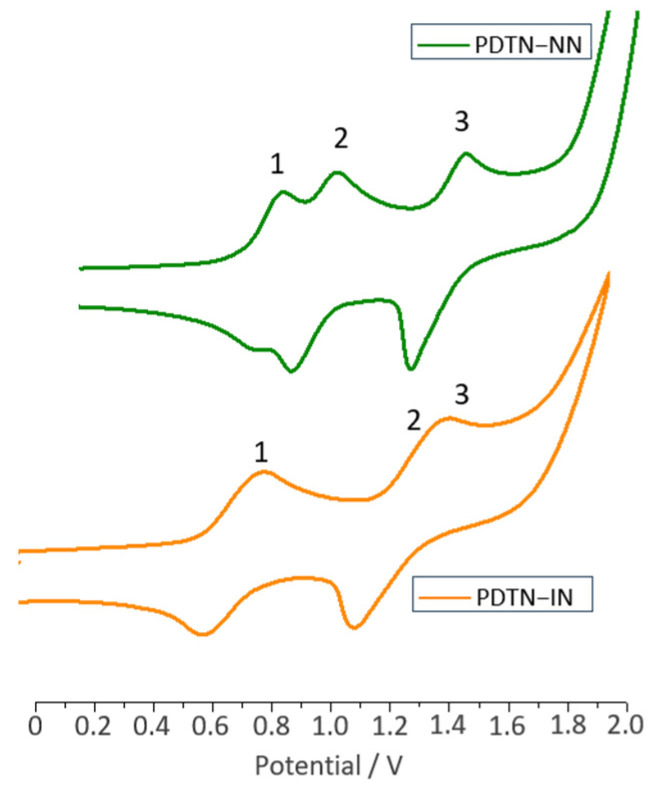
The cyclic voltammetry curves of PDTN-NN (upper) and PDTN-IN (lower) recorded in DCM at room temperature, respectively (Bu_4_NPF_6_, 0.1 M, the scan rate is 100 mV/s).

CV experiments were performed by using a three-electrode cell in DCM solution of tetrabutylammonium tetrafluoroborate (n-C_4_H_9_)_4_NPF_6_ (0.1 M) with a scan rate of 100 mV/s at room temperature. CV curves (the oxidation part) of PDTN-NN and PDTN-IN are shown in Figure 3. The redox process of the NN radical was reversible under the CV measurement conditions. In contrast, the IN radical exhibited a similar reduction cycle but an irreversible oxidation cycle, with its oxidation potential approximately 0.32 V higher than that of the NN group, lacking a complete oxidation peak. When organic radicals are connected to phenothiazine and subjected to CV experiments, the curve exhibits multiple oxidation peaks, indicating the generation of new radical species during the oxidation process.

**Scheme 4:**
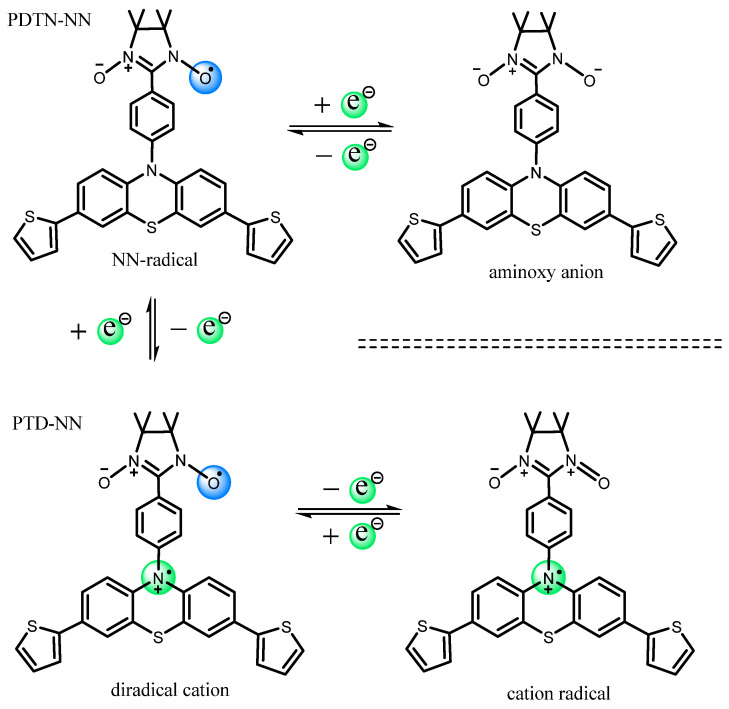
The electron transfer among four redox states in CV measurements.

We selected PDTN-NN as a representative example to analyze the three oxidation potentials in detail (Scheme 4). The first oxidation peak appeared at E_Oxi-1_ = 0.78 V, indicating the loss of one electron from the tertiary amino group, simultaneously forming a new nitrogen cation radical. At this stage, the entire molecule PDTN-NN transformed into a diradical cation (PTD-NN). Subsequently, the second oxidation at E_Oxi-2_ = 0.93 V caused the NN group to lose an electron, converting it into an oxoammonium cation. As a result, the entire molecule PTD-NN transitioned into cation monoradical. The third oxidation peak, observed at E_Oxi-3_ = 1.37 V, corresponded to the conversion of the cation radical into a dication, with no remaining unpaired electrons on the molecular backbone. Through the analysis of oxidation potential and electron transfer, it can be confirmed that the redox process generates diradical cations and cation radical by CV measurements. Indeed, the oxidation potential of phenothiazine is lower than that of the NN and IN groups. This oxidation sequence provides the necessary conditions for the formation of cation diradical [1]. For PDTN-IN, the positions of the first and third oxidation peaks are nearly identical to those of PDTN-NN, as shown in Figure 3 (orange spectrum). However, the second oxidation peak shifted to E_Oxi-2_ = 1.25 V due to the higher oxidation potential of the IN group. The CV spectra of PO-NN and PO-IN are presented in Figure S5. The third oxidation peak was absent because the second electron oxidation potential of phenoxazine exceeded E_Oxi_ = 1.9 V, which was beyond the measurement range. For comparison, we also measured the CV oxidation spectra of PTP, PDTN and BP-NN in Figure S6. All oxidation potential peaks are summarized in Table 1.

### 2.3. EPR Measurements and Properties

#### 2.3.1. EPR Spectra of Redox Stimuli Responsive Process

**Figure 4:**
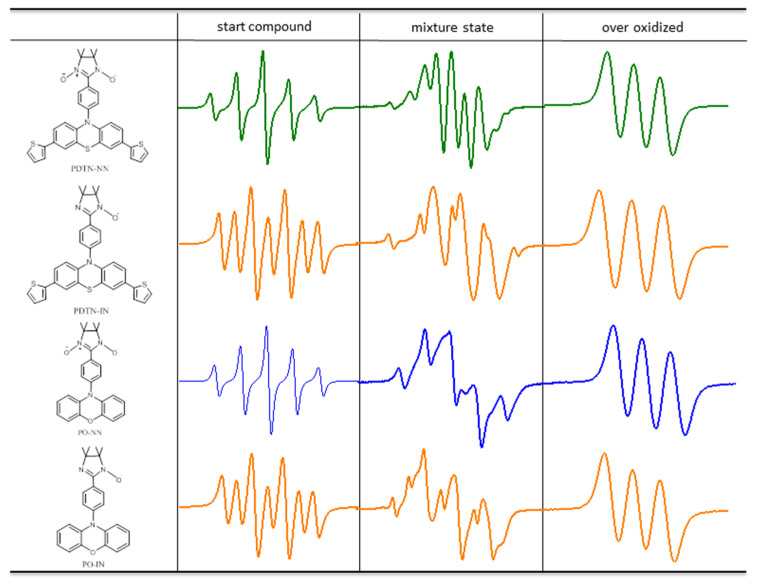
EPR spectra curves of PDTN-NN, PDTN-IN, PO-NN and PO-IN with different oxidation states (representative EPR spectra curves which contain biradical features were chosen for mixture state) in DCM (c~10^−4^ M) at room temperature.

The representative spectra of starting compound, mixture state and over-oxidized about the four monoradicals during the whole oxidation process are shown in Figure 4. The starting compound exhibited original quintet (the EPR spectrum of PDTN-NN with g = 2.0071, a_N_ = 7.67 G; the EPR spectrum of PO-NN with g = 2.0071, a_N_ = 7.72 G) and septet (the EPR spectrum of PDTN-IN with g = 2.0064, a_N1_ = 4.51 G and a_N2_ = 9.02 G; the EPR spectrum of PO-IN with g = 2.0063, a_N1_ = 4.25 G and a_N2_ = 8.51 G) signal patterns due to the influence of NN and IN radicals. Under the influence of hyperfine splitting from the newly formed nitrogen radical cation, the number of peaks in the EPR spectrum of the mixture state increased due to further splitting. Upon reaching the over-oxidized state, the EPR signal resembled a typical nitrogen cation monoradical, characterized by a three-line signal pattern. The g factors of spectra for each state are listed in Table 2.

**Figure 5:**
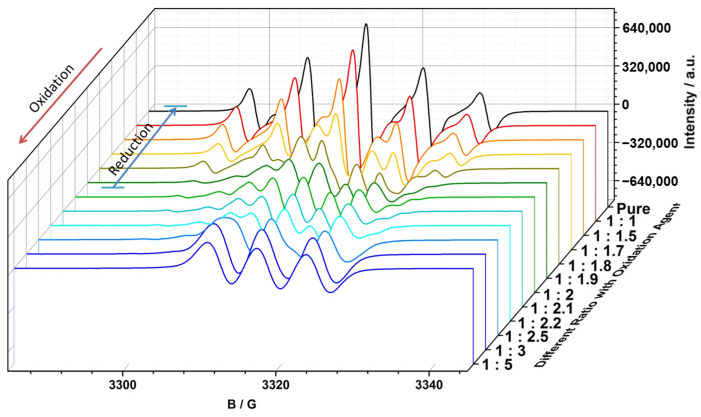
X-band EPR spectra of PDTN-NN with different ratios of oxidant SbCl_5_ (during the titration of oxidation process) in DCM (~10^−4^ M) at room temperature.

We conducted a detailed titration experiment of PDTN-NN for EPR measurement to check the redox-responsive radical behavior by a series ratio of oxidant SbCl_5_ and one reducer Zn powder. Some special points of oxidation titration as representative of different oxidation states are chosen and listed in Figure 5. Based on the data and analysis of EPR, the molecule exhibited changes in radical structures and spin switch during the oxidation process. When the ratio of oxidant to monoradical reached 2:1, the line pattern of spectrum changed from an initially symmetrical five-line gradually to a symmetrical nine-line, indicating the emergence of a new superimposed symmetrical seven-line spectrum. The change in the spectral peaks confirms the existence of a new radical species, the diradical cation, during the oxidation process. More NN groups were decomposed as the oxidation progressed, and the spectrum of nine-line pattern gradually changed to three-line pattern (typical nitrogen cation monoradical).

**Figure 6:**
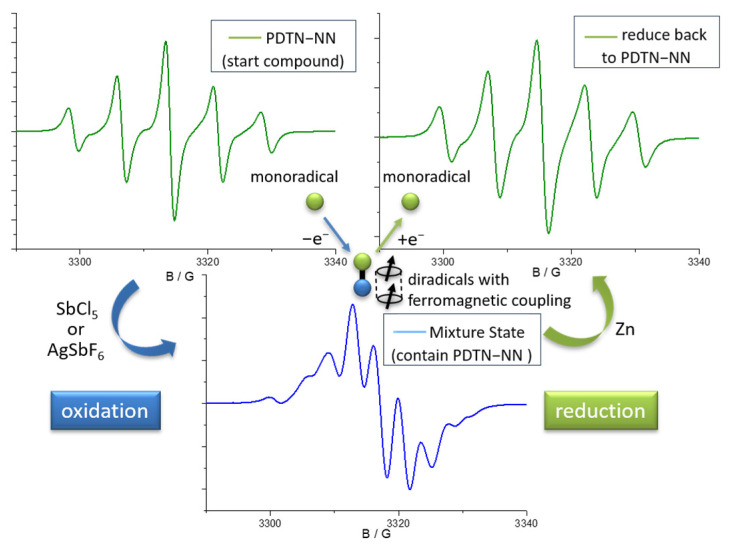
The EPR spectra of reversible transition of bidirectional oxidation–reduction process for PDTN-NN.

We made reduction experiments which are the reverse process of oxidation titration with the reductant Zn to check whether the stimuli responsive process is reversible. Before the formation of the over-oxidized compound, the mixture state could be reversibly reduced back to pure PDTN-NN, exhibiting a five-line signal pattern. However, once over-oxidized, the nitrogen cation monoradical could not be reduced back to PDTN-NN. This demonstrates that PTD-NN can be reversibly reduced to pure PDTN-NN. The circular transformation of the redox process by oxidation and reduction is shown in Figure 6. Depending on the EPR spectra of bidirectional switch, we conclude that the transformation of the redox-responsive radical behavior is a reversible process under suitable oxidative conditions. We simulated the EPR spectra of the starting compound, the over-oxidized state and PTD-NN, as shown in Figure S8. They both perfectly fit with the experimental data, which are shown above. We also performed the oxidation process of PTP, PDTN and BP-NN for EPR measurement with oxidant SbCl_5_ in Figures S9 and S10, and the results obtained were consistent with the predictions.

#### 2.3.2. Fitting of Simulated EPR with New Signal Pattern of Experimental EPR Spectra

**Figure 7:**
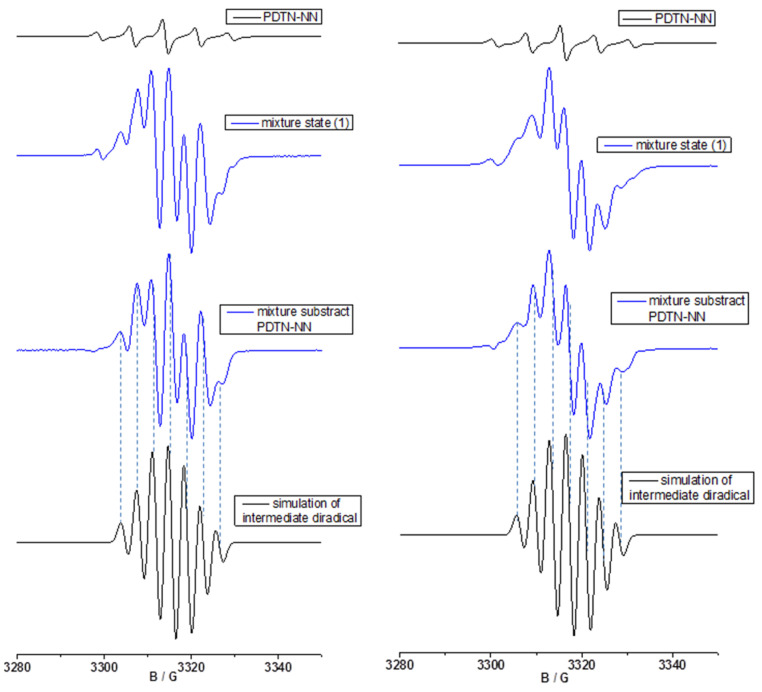
Experimental EPR spectra and simulated EPR spectra analysis of the first mixture state for PDTN-NN by the situation of oxidant SbCl_5_ (**left**), and the situation of oxidant AgSbF_6_ (**right**). The dotted lines are used to indicate the correspondence between two spectra.

To demonstrate the formation of diradical cations during the oxidation process, we compared the experimental spectra with the simulated spectra for three different mixture states. The EPR spectra and the first mixture state analysis of PDTN-NN in the case of oxidant SbCl_5_ (left) and oxidant AgSbF_6_ (right) are shown in Figure 7. Two black spectra represent the experimental spectra of the pure PDTN-NN (g = 2.0071, a_N_ = 7.67 G) and the simulated spectra of pure PTD-NN (g = 2.0063, a_N1_ = 3.76 G, a_N2_ = 3.25 G), as shown in the left panel, corresponding to standard five-line spectrum and seven-line spectrum. The first blue spectrum represents the experimental spectrum of the first mixture state, exhibiting an approximately nine-line hyperfine splitting pattern. All spectra were plotted in the same figure with a consistent magnetic field range selected for comparison. In the first mixture state, the first peak of the mixture spectrum perfectly matches in both position and shape with the pure PDTN-NN spectrum, confirming that this peak originates from the contribution of the monoradical. By adjusting the signal intensity of the PDTN-NN spectrum, we ensured that the influence of the monoradical was completely removed, resulting in a new subtracted spectrum. Comparing with the black simulated standard seven-line spectrum of PTD-NN, we found that patterns, numbers, positions and the relative intensity of the two spectral splitting peaks corresponded, indicating that the target product diradical cation (composed of a stable neutral NN radical and a nitrogen cation radical formed by charge transfer through the oxidation of the tertiary nitrogen on the conjugated phenothiazine structure) was successfully formed during the oxidation process. We selected a different oxidant AgSbF_6_ and employed the same methodology in the right panel, obtaining identical results. The evidence suggests that during the oxidation of PDTN-NN using two different oxidants, diradical cation exists in both cases. It also indirectly proves that the newly generated diradical spin species is not a specific spin signal induced by a particular oxidant such as SbCl_5_, but rather by a normal and universal oxidation process of charge transfer.

**Figure 8:**
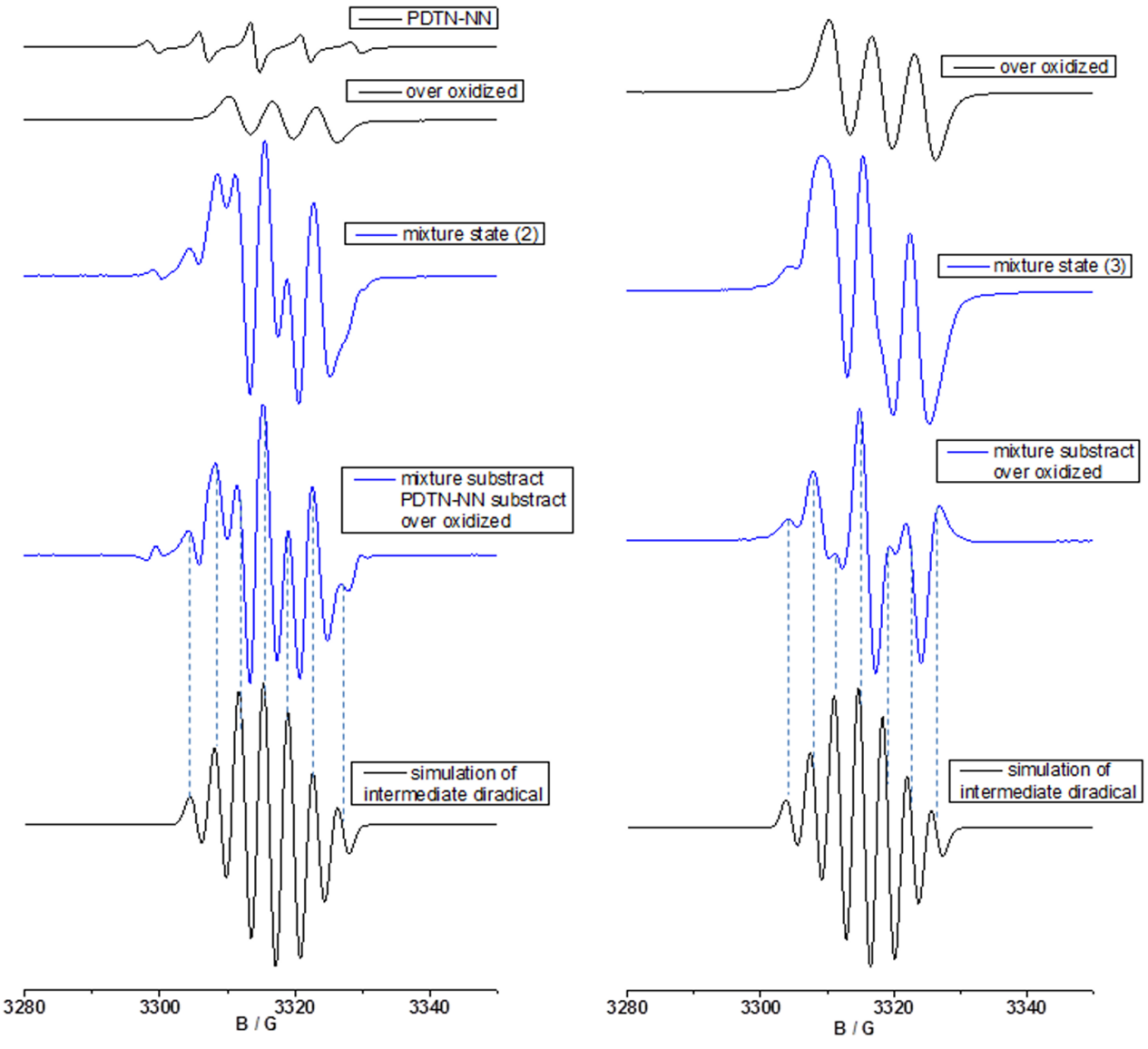
Experimental EPR spectra and simulated EPR spectra analysis of the second mixture state for PDTN-NN (**left**) and the third mixture state for PDTN-NN (**right**) by the situation of oxidant SbCl_5_. The dotted lines are used to indicate the correspondence between two spectra.

We selected the same oxidation SbCl_5_ and employed the same method to analyze the spectra of the second and third mixture states in Figure 8. The spectral analysis of the second mixture state is presented in the left panel of Figure 8. Due to the oxidation and degradation of a portion of the NN radicals, nitrogen cation monoradical was generated in the second mixture state. So, we simulated the spectrum of the pure nitrogen cation monoradical (g = 2.0052, a_N_ = 6.5 G), which exhibits a distinct three-line pattern. The EPR spectrum of the second mixture state is influenced by both PDTN-NN and the over-oxidized species. We adjusted the radical signal with appropriate parameters and subtracted the background contribution to obtain a new subtracted spectrum. Comparing with the simulated diradical cation spectrum, we found that patterns, numbers, and positions of the new spectrum also matched well to the simulated seven-line spectrum, which proves the formation of diradical cation.

As the proportion of the oxidant continuously increased, the NN group could not withstand such a high concentration of the oxidant and decomposed. At this stage, no pure PDTN-NN remained. The spectral analysis of the third mixture state is presented in the right panel of Figure 8. We clearly observed that the first peak in the mixture spectrum was different from that of the over-oxidized state spectrum. After subtracting the symmetric over-oxidized state spectrum from the experimental spectrum, a new spectrum was obtained and compared with the simulated spectrum. Although the peak shapes of the new spectrum and the simulated seven-line spectrum were significantly different, the positions of each splitting peak aligned well between the two spectra. According to the analysis results, we speculate that as the oxidation process progresses to the third mixed state, it primarily consists of over-oxidized species with only a trace amount of diradical cation, leading to significant differences when compared to the simulated spectrum. Through the analysis of the three mixture states, it provides convincing EPR evidence for the presence of diradical cation during the oxidation process.

### 2.4. DFT Calculations of Redox Stimuli Responsive System

**Figure 9:**
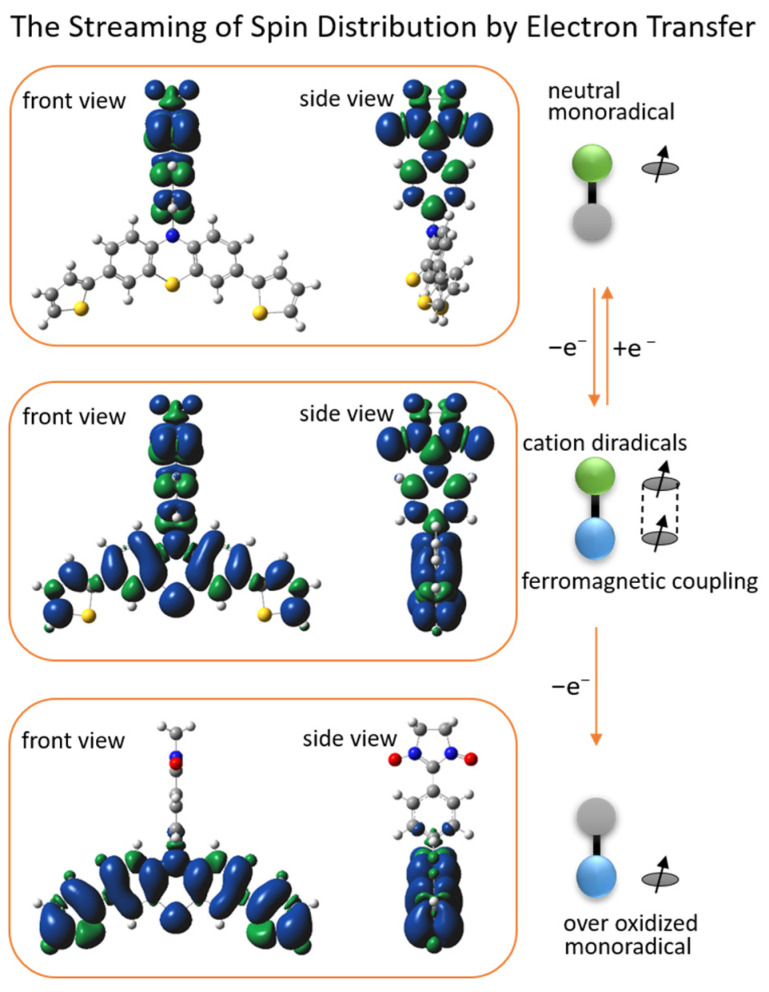
The spin density distribution of PDTN-NN among different states (neutral monoradical, diradical cation and over-oxidized cation monoradical) calculated by DFT (Gaussian 09W).

**Figure 10:**
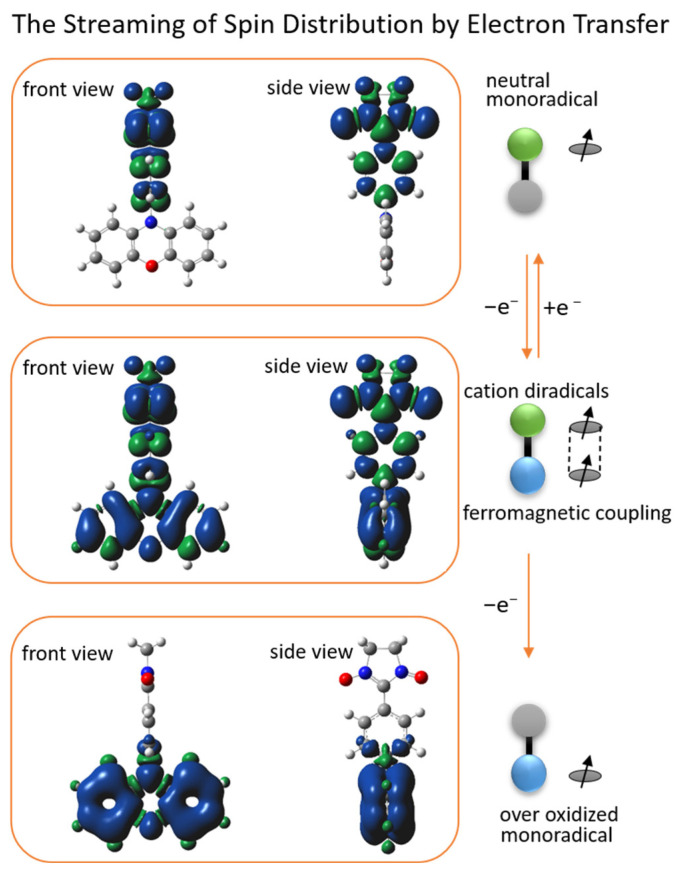
The spin density distribution of PO-NN among different states (neutral monoradical, diradical cation and over-oxidized cation monoradical) calculated by DFT (Gaussian 09W).

DFT (density functional theory) Gaussian 09W was used to calculate the spin density distribution and the magnetic exchange coupling constant (J) of PDTN-NN and PO-NN with three different redox states. The Optimization Plus Frequency method and the UBLYP/6-31G(d) basis set were used for these calculations. The front and side views of the spin density distributions for the three radical species of PDTN-NN and PO-NN are illustrated in Figure 9 and Figure 10, respectively. It was observed that the spin distributions of PDTN-NN and PO-NN were concentrated at the range of NN group, while the spin distributions of PTD-NN and POD-NN were delocalized in whole molecules. At the over-oxidized state, the spin density distributions were concentrated at the range of phenothiazine group and phenoxazine group. The changes in spin distribution by the electron transfer in redox reactions were clearly demonstrated by the molecular backbone. This confirms that we can controllably regulate the intramolecular spin-polarized flow through external redox stimuli, thereby altering its intramolecular spin characteristics and magnetic properties. According to the spin density distribution, it is observed that the newly formed diradical cation generated by oxidation possesses two spin centers. First, we predict that the diradical cation exhibits intramolecular ferromagnetic interactions based on the spin polarization rule. The DFT calculations yielded the coupling constant (*J* = 1168.6 cm^−1^) for PTD-NN and the coupling constant (*J* =340.2 cm^−1^) for POD-NN, with the relevant data provided in Table S2. The calculated values are within the range of DFT results observed for similar systems [1,22,43]. The positive *J* values indicate that both diradical cations tend to exhibit intramolecular ferromagnetic exchange interactions. Additionally, EPR measurements were conducted at 120 K in DCM. In the forbidden transition (Δms = 2) region around g ≈ 4, distinct signals were detected for PTD-NN (upper panel) and POD-NN (lower panel) in Figure S11. This provides experimental evidence of intramolecular spin coupling.

**Figure 11:**
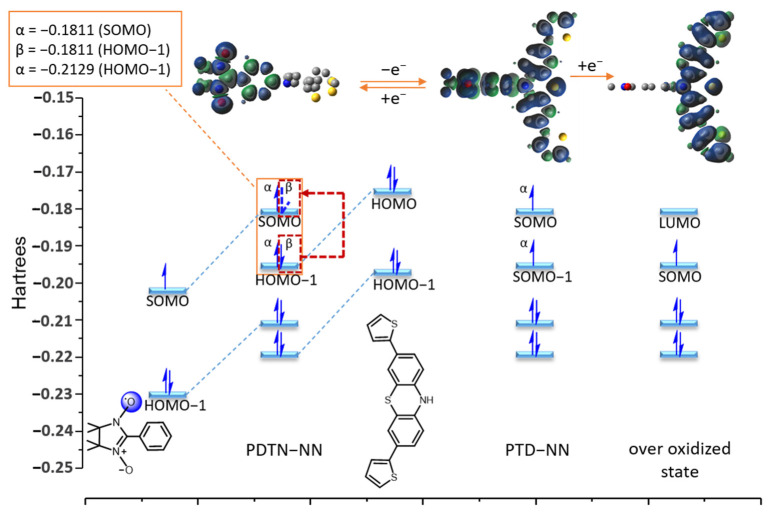
The SOMO, HOMO and LUMO energy levels and electron distributions on orbitals for different oxidation states of PDTN-NN, phenyl-NN and dithiophen-phenothiazine.

DFT calculations were used to simulate the frontier orbital energies (SOMO, HOMO, and LUMO) and electron distributions in these orbitals for different oxidation states of PDTN-NN. The calculations used the same basis set, and the results are shown in Figure 11. The structure of PDTN-NN was divided into two parts: the open-shell molecule phenyl-NN and the closed-shell molecule dithiophen-phenothiazine. Their frontier orbital energy and outer electron distributions are also displayed in Figure 11. For PDTN-NN, the energy of the α electron on SOMO is −0.1811 Hartrees, and the energy of the α and β electrons on HOMO−1 is −0.2129 Hartrees and −0.1811 Hartrees. Due to energy degeneracy forming a metastable state, the β-electron on HOMO−1 can transfer to SOMO through spin streaming. So, the oxidation process involving the loss of one electron is likely to occur from the β electron on HOMO−1. We obtained the frontier orbital energy and outer electron distributions of PTD-NN, which exhibit intramolecular coupling interactions. PTD-NN is further oxidized to the over-oxidized state, where only one spin center remains within the molecule. Consequently, spin distribution streaming is observed to propagate through the entire molecular backbone.

## 3. Materials and Methods

All reagents required for the synthesis were purchased from Aladdin and Energy Chemical and used in accordance with the specified requirements. Prior to the experiments, all reaction vessels were dried for use and the experiments were carried out under the protection of an argon atmosphere. The compound 2,3-bis(hydroxyamino)-2,3 dimethylbutane (BHA) was synthesized following a procedure outlined in the literature [44]. The specific synthesis details can be found in the Supplementary Materials.

Initially, the secondary amine moieties in phenothiazine and phenoxazine were coupled with the boronic acid group by following Chan–Lam coupling reaction [45,46,47]. The successful introduction of an aldehyde group was a crucial step in the entire synthetic pathway. Next, the aldehyde group was subjected to Ullmann condensation with the previously synthesized BHA, followed by oxidation to obtain the desired radical. The target products were separated by column chromatography. The characterization of compounds was performed using NMR spectroscopy (Figure S1). The electronic interactions between donor groups and nitroxide radicals were verified by UV-Vis absorption spectroscopy and cyclic voltammetry. The whole process of spin characteristics and magnetic properties of four synthesized paramagnetic molecules were investigated by using EPR. DFT calculations were performed to determine the electron spin density distributions of organic radicals in different valence states and to obtain the intramolecular coupling constants of the diradical cations.

## 4. Conclusions

We successfully synthesized four monoradicals, namely PDTN-NN, PDTN-IN, PO-NN and PO-IN, which could be oxidized to their corresponding diradical cations. This work demonstrates the potential of utilizing redox stimuli to modulate spin characteristics and magnetic properties. UV-Vis absorption spectroscopy, cyclic voltammetry and EPR were used to analyze and characterize the entire oxidation process. The flow of spin density distribution throughout the molecule was clearly displayed by DFT calculations. The synthesized diradical cations were confirmed to exhibit intramolecular coupling interactions. In addition, the redox stimuli response process of paramagnetic molecules is demonstrated to be reversible. These spin system characteristics hold promising potential for applications in spintronics [48,49,50,51].

## Figures and Tables

**Table 1:** The different oxidation potentials of the molecules.

Potential [V]	PTP	PDTN	BP-NN	PDTN-NN	PDTN-IN	PO-NN	PO-IN
E_Oxi-1_	0.63	0.61	0.91	0.78	0.68	0.77	0.62
E_Oxi-2_	1.42	1.25	—	0.93	1.25	0.91	1.23
E_Oxi-3_	—	—	—	1.37	1.28	—	—

**Table 2:** The corresponding g factor of PDTN-NN, PDTN-IN, PO-NN and PO-IN in different oxidation states.

G Factor	Starting Compound	Mixture State	Over-Oxidized	Diradical Cation
PDTN-NN	2.0071	2.0067	2.0053	2.0063
PDTN-IN	2.0064	2.0062	2.0052	2.0059
PO-NN	2.0071	2.0064	2.0034	2.0056
PO-IN	2.0063	2.0058	2.0035	2.0052

## Data Availability

Data are contained within the article and Supplementary Materials.

## Supplementary Materials

The following supporting information can be downloaded at: https://www.mdpi.com/article/10.3390/molecules30091931/s1, Scheme S1: Synthesis of cation monoradicals PTPC, PDTC and bromobenzene-NN; Figure S1: ^1^H NMR spectrum of 3; Figure S2: UV-Vis absorption spectra of the whole oxidation titration process of PDTN-IN; Figure S3: UV-Vis absorption spectra of the whole oxidation titration process of PO-NN; Figure S4: UV-Vis absorption spectra of the whole oxidation titration process of PO-IN; Figure S5: The cyclic voltammetry curves of PO-NN, PO-IN; Figure S6: The cyclic voltammetry curves of PTP, PDTN, and BP-NN; Figure S7: The first oxidation and reduction of cyclic voltammetry curves of PDTN-NN and PDTN-IN; Figure S8: The simulated EPR spectra of (a) PDTN-NN, (b) the over-oxidized state, (c) the mixture of (a) and (b) with the ratio 1: 1, (d)PTD-NN; Figure S9: X-band EPR spectra of BP-NN with different ratios of oxidant; Figure S10: X-band EPR spectra of PTP, PDTN and the oxidized cation monoradicals PTPC and PDTC; Figure S11: the peak signal appears both in PTD-NN sample (upper) and POD-NN sample (lower) in the forbidden transition (Δms = 2) around g ≈ 4 region, measured at 120 K in DCM; Figure S12: EPR Spectra of Intermediate states of PO-NN and PO-IN with temperature dependent reversible features in DCM; Table S1: The optical and electrochemical energy levels of PDTN-NN and PDTN-IN; Table S2: Summary of diradical cations calculation results.

## References

[1] Kolanji K., Baumgarten M. (2020). Dithienopyrrole Derivatives with Nitronyl Nitroxide Radicals and Their Oxidation to Cation High-Spin Molecules. Chem. Eur. J..

[2] Yokoyama N., Tanaka N., Fujimoto N., Tanaka R., Suzuki S., Shiomi D., Sato K., Takui T., Kozaki M., Okada K. (2021). Syntheses and Properties of (Nitronyl nitroxide)-substituted Triphenylamine ortho-Bridged by Two Oxygen and Sulfur Atoms. Chem. Asian J..

[3] Kuratsu M., Suzuki S., Kozaki M., Shiomi D., Sato K., Takui T., Kanzawa T., Hosokoshi Y., Lan X.Z., Miyazaki Y. (2012). (Nitronyl Nitroxide)-Substituted Trioxytriphenylamine Radical Cation Tetrachlorogallate Salt: A 2p-Electron-Based Weak Ferromagnet Composed of a Triplet Diradical Cation. Chem. Asian J..

[4] Tretyakov E.V., Ovcharenko V.I. (2009). The Chemistry of Nitroxide Radicals in the Molecular Design of Magnets. Rus. Chem. Rev..

[5] Ionita P., Whitwood A.C., Gilbert B.C. (2001). Synthesis and Characterization of Some Novel Hetero-diradicals Containing Linked Hydrazyl and Aminoxyl (Nitroxide) Moieties. J. Chem. Soc. Perkin Trans..

[6] Ratera I., Veciana J. (2012). Playing with Organic Radicals as Building Blocks for Functional Molecular Materials. Chem. Soc. Rev..

[7] Wei D.D., Qin Y.L., Xu Z.P., Liu J., Chen R.R., Yu Y., Wang D. (2024). Study of Molecular Dimer Morphology Based on Organic Spin Centers: Nitronyl Nitroxide Radicals. Molecules.

[8] Yue Z., Liu J., Baumgarten M., Wang D. (2023). Spirobifluorene Mediating the Spin−Spin Coupling of Nitronyl Nitroxide Diradicals. J. Phys. Chem. A.

[9] Wang D., Ma Y.J., Wolf B., Kokorin A.I., Baumgarten M. (2018). Temperature-Dependent Intramolecular Spin Coupling Interactions of a Flexible Bridged Nitronyl Nitroxide Biradical in Solution. J. Phys. Chem. A.

[10] Wang D., Shi C.F., Baumgarten M., Wang W.P., Liu J. (2024). Nonalternating π-System Mediated Spin Coupling in Azulene Nitronyl Nitroxide Diradicals. J. Org. Chem..

[11] Luo J.S., Wan Z.Q., Jia C.Y. (2016). Recent advances in phenothiazine-based dyes for dye-sensitiezed solar cells. Chin. Chem. Lett..

[12] Wainwright M. (2015). Tricyclic Cation Chromophores as Models for New Photoantimicrobials. J. Braz. Chem. Soc..

[13] Massie S.P. (1954). The Chemistry of Phenotiazine. Chem. Rev..

[14] Opperman K.A., Mecklenburg S.L., Meyer T.J. (1994). Intramolecular, Photoinduced Electron Transfer in Ruthenium(II) Bipyridine-Quinone Complexes. Inorg. Chem..

[15] Shruti, Dwivedi J., Kishore D., Sain S. (2018). Recent Advancement in The Synthesis of Phenoxazine Derivatives and Their Analogues. Synth. Commun..

[16] Zheng X., Wang S.Y., Qiu Y.F., Li Y.T., Zhou C.K., Sui Y.X., Ma J., Wang X.P. (2013). One-Electron Oxidation of an Organic Molecule by B(C_6_F_5_)_3_; Isolation and Structures of Stable Non-para-Substituted Triarylamine Cation Radical and Bis(triarylamine) Dication Diradicaloid. J. Am. Chem. Soc..

[17] Oka H. (2010). Synthesis and Magnetic Properties of Orthogonally Linked Phenothiazing Cation Radical Dimer and Tetramer. Org. Lett..

[18] Okada K., Imakura T., Oda M., Murai H. (1996). 10,10′-(m- and p-Pheny-lene)diphenothiazine Dications: Violatiion of a Topology Rule in Heterocyclic High-Spin *π*-Systems. J. Am. Chem. Soc..

[19] Okamoto T., Kuratsu M., Kozaki M., Hirotsu K., Ichimura A., Matsushita T., Okada K. (2004). Remarkable Structure Deformation in Phenothiazaine Trimer Radical Cation. Org. Lett..

[20] Kuratsu M., Kozaki M., Okada K. (2005). 2,2′:6′2′′:6′′,6-Trioxytriphenylamine: Synthesis and Properties of the Radical Cation and Neutral Species. Angew. Chem. Int. Ed..

[21] Suzuki S., Nakamura F., Naota T. (2018). Direct Synthetic Method for (Nitronyl Nitroxide)-substituted π-Electronic Compounds by Palladium-catalyzed Cross-Coupling Reaction with a Zinc Complex. Mater. Chem. Front..

[22] Tahara T., Suzuki S., Kozaki M., Shiomi D., Sugisaki K., Sato K., Takui T., Miyake Y., Hosokoshi Y., Nojiri H. (2019). Triplet Diradical Cation Salts Consisting of Phenothiazine Radical Cation and Nitronyl Nitroxide. Chem. Eur. J..

[23] Kuratsu M., Suzuki S., Kozaki M., Shiomi D., Sato K., Takui T., Okada K. (2007). Magnetic Interaction of Tri- and Di-oxytriphenylamine Radical Cation FeCl_4_ Salts. Inorg. Chem..

[24] Suzuki S., Nagata A., Kuratsu M., Kozaki M., Tanaka R., Shiomi D., Sugisaki K., Toyota K., Sato K., Takui T. (2012). Trinitroxide-Trioxytriphenylamine: Spin-State Conversion from Triradical Doublet to Diradical Cation Triplet by Oxidative Modulation of a p-Conjugated System. Angew. Chem. Int. Ed..

[25] Sato O., Tao J., Zhang Y.Z. (2007). Control of Magnetic Properties through External Stimuli. Angew. Chem. Int. Ed..

[26] Nakatsuji S. (2004). Recent Progress toward the Exploitation of Organic Radical Compounds with Photo-Responsive Magnetic Properties. Chem. Soc. Rev..

[27] Jalilov A.S., Han L., Nelsen S.F., Guzei I.A. (2013). Oxidation Products of Doubly Trimethylene-Bridged Tetrabenzyl p-Phenylenediamine Paracyclophane. J. Org. Chem..

[28] Nelsen S.F., Ismagilov R.F., Teki Y. (1998). Comparison of the Singlet, Triplet Energy Gap of a Symmetrical Diradical Dication with ET Parameters Derived from Its Optical Spectrum. J. Am. Chem. Soc..

[29] Barlow S., Risko C., Odom S.A., Zheng S.J., Coropceanu V., Beverina L., Brédas J.L., Marder S.R. (2012). Tuning Delocalization in the Radical Cations of 1,4-Bis[4(diarylamino)styryl]benzenes, 2,5-Bis[4(diarylamino)styryl]thiophenes, and 2,5-Bis[4(diarylamino)styryl]pyrroles through Substituent Effects. J. Am. Chem. Soc..

[30] Sato K., Yano M., Furuichi M., Shiomi D., Takui T., Abe K., Itoh K., Higuchi A., Katsuma K., Shirota Y. (1997). Polycation High-Spin States of One- and Two-Dimensional (Diarylamino)benzenes, Prototypical Model Units for Purely Organic Ferromagnetic Metals As Studied by Pulsed ESR/ Electron Spin Transient Nutation Spectroscopy. J. Am. Chem. Soc..

[31] Stickley K.R., Blackstock S.C. (1994). Triplet Dication and Quartet Trication of a Triaminobenzene. J. Am. Chem. Soc..

[32] Bushby R.J., McGill D.R., Ng K.M., Taylor N. (1997). Disjoint and coextensive diradical diions. J. Chem. Soc. Perkin Trans. 2.

[33] Yokoyama Y., Sakamaki D., Ito A., Tanaka K., Shiro M. (2012). A Triphenylamine Double-Decker: From a Delocalized Radical Cation to a Diradical Dication with an Excited Triplet State. Angew. Chem. Int. Ed..

[34] Fatila E.M., Clérac R., Rouzières M., Soldatov D.V., Jennings M., Preuss K.E. (2013). High-Spin Ribbons and Antiferromagnetic Ordering of a MnII-Biradical-MnII Complex. J. Am. Chem. Soc..

[35] Karpinska J., Starczewska B., Tarasiewicz H.P. (1996). Analytical Properties of 2- and 10-Disubstituted Phenothiazine Derivatives. Anal. Sci..

[36] Baumgarten M., Gügel A. (1993). EPR and Optical Absorption Spectra of Reduced Buckminsterfullerene. Adv. Mater..

[37] Rathore R., Burns C.L. (2003). A Practical One-Pot Synthesis of Soluble Hexa-peri-hexabenzocoronene and Isolation of Its Cation-Radical Salt. J. Org. Chem..

[38] Rosspeintner A., Griesser M., Matsumoto I., Teki Y., Li G.Q., Nelsen S.F., Gescheidt G. (2010). EPR and ENDOR Studies of Dimeric Paracyclophane Radical Cations and Dications Containing Tri- and Pentamethylene-Bridged p-Phenylene Diamine Units. J. Phys. Chem. A..

[39] Nelsen S.F., Ismagilov R.F., Powell D.R. (1997). Charge-Localized p-Phenylenedihydrazine Radical Cations: ESR and Optical Studies of Intramolecular Electron Transfer Rates. J. Am. Chem. Soc..

[40] Maurel V., Skorka L., Onofrio N., Szewczyk E., Djurado D., Dubois L., Mouesca J.M., Kulszewicz-Bajer I. (2014). Ferromagnetic Spin Coupling through the 3,4′-Biphenyl Moiety in Arylamine Oligomers Experimental and Computational Study. J. Phys. Chem. B.

[41] Fang S., Lee M.S., Hrovat D.A., Borden W.T. (1995). Ab Initio Calculations Show Why m-Phenylene Is Not Always a Ferromagnetic Coupler. J. Am. Chem. Soc..

[42] Mañeru D.R., Pal A.K., Moreira I.P.R., Datta S.N., Illas F. (2014). The Triplet−Singlet Gap in the m-Xylylene Radical: A Not So Simple One. J. Chem. Theory Comput..

[43] Hiraoka S., Okamoto T., Kozaki M., Shiomi D., Sato K., Takui T., Okada K. (2004). A Stable Radical-Substituted Radical Cation with Strongly Ferromagnetic Interaction:  Nitronyl Nitroxide-Substituted 5,10-Diphenyl-5,10-dihydrophenazine Radical Cation. J. Am. Chem. Soc..

[44] Ovcharenko V.I., Fokin S.V., Romanenko G.V., Korobkov I.V., Rey P. (1999). Synthesis of vicinal bishydroxylamine. Russ. Chem. Bull..

[45] Rao K.S., Wu T.S. (2012). Chan-Lam Coupling Reactions: Synthesis of Heterocycles. Tetrahedron.

[46] Chan D.M.T., Monaco K.L., Wang R.P., Winters M.P. (1998). New N- and O-Arylations with Phenylboronic Acids and Cupric Acetate. Tetrahedron Lett..

[47] Wexler R.P., Nuhant P., Senter T.J., Gale-Day Z.J. (2019). Electrochemically Enabled Chan–Lam Couplings of Aryl Boronic Acids and Anilines. Org. Lett..

[48] Guo H.Q., Peng Q.M., Chen X.K., Gu Q.Y., Dong S.Z., Evans E.W., Gillett A.J., Ai X., Zhang M., Credgington D. (2019). High stability and luminescence efficiency in donor–acceptor neutral radicals not following the Aufbau principle. Nature Materials.

[49] Dong X., Luo Q.C., Zhao Y., Wang T., Sun Q.C., Pei R.B., Zhao Y., Zheng Y.Z., Wang X. (2023). A Dynamic Triradical: Synthesis, Crystal Structure, and Spin Frustration. J. Am. Chem. Soc..

[50] Bras T., Hsu C., Baum T.Y., Vogel D., Mayor M., van der Zant H.S.J. (2025). Mechanically Stable Kondo Resonance in an Organic Radical Molecular Junction. J. Phys. Chem. C.

[51] Lang Y.R., Hei Y.X., Yu T.R., Yang Y.T., Chen M., Zhang J.R., Zhao F.G., Zheng Y.H., Liu X.S. (2024). A donor–acceptor coupling unit modulates the spin coupling effect of a stable diradical. J. Mater. Chem. C.

