# Supplementary material for: Oxidation-Triggered Formation of Diradical Cations from Paramagnetic Molecules and Their Spin Density Evolution
Source: Molecules. 2025 Apr 26;30(9):1931. doi: 10.3390/molecules30091931 (PMC12073237; doi:10.3390/molecules30091931)
Supplement: Supplementary file 1 [file molecules-30-01931-s001.zip › molecules-3554522-SI.pdf]

Oxidation-Triggered Formation of Diradical Cations from Paramagnetic Molecules and Their Spin Density Evolution

**Di Wang [1,*], Dan Yao [1], Xinyu Li [1], Lingli Shi [1], Chunyuan Wang [1], Jie Li [1], Weili Kong [1], Yongliang Qin [2,*]
and Martin Baumgarten [3]**

[1] School of Materials Science and Chemical Engineering, Anhui Jianzhu University, Hefei 230601, China; 19719270397@163.com (D.Y.); lxy954003@163.com (X.L.); 15856948859@163.com (L.S.); 19855119474@163.com (C.W.); ljsunness@126.com (J.L.); kwl123@ahjzu.edu.cn (W.K.)
[2] Anhui Province Key Laboratory of Condensed Matter Physics at Extreme Conditions, Hefei Institutes of Physical Science, Chinese Academy of Sciences, Hefei 230031, China
[3] Max Planck Institute for Polymer Research, Mainz 55128, Germany; baumgart@mpip-mainz.mpg.de
* Correspondence: wangdi@ahjzu.edu.cn (D.W.); ylqin@ipp.ac.cn (Y.Q.)

Contents

Materials. **S1**

Synthesis **S1-10**

Supporting NMR spectra **S11**

Supporting UV-Vis Absorption Spectra **S12-13**

Supporting CV Measurements **S14-16**

EPR spectra **S17-21**

DFT Calculations **S22**

Materials

Unless otherwise noted, the UV-Vis absorption spectra were recorded on a Shanghai Jingke L7 spectrophotometer with dichloromethane (c ~ 10^{-5} M) as the solvents for the experiments at room temperature. IR spectra were recorded in a Nicolet 730 FT-IR spectrometer at room temperature. Proton nuclear magnetic resonance (1H NMR) spectra were recorded on a 250 MHz Bruker spectrometer with DMSO-*d*6 and CD_2Cl_2 as the solvent for proton magnetic resonance (1H NMR) spectra, and the data are reported in ppm relative to the internal standard Me_4Si. Mass spectra were obtained on FDMS VG Instruments ZAB-2 mass spectrometer. All electrochemical measurements were performed using CHI760E (Shanghai Chenhua, China). EPR spectra were recorded in dilute oxygen-free solutions of dichloromethane (c ~ 10^{-4} M) by using a Bruker EMX-plus spectrometer equippedwith an NMR gauss meter (Bruker ER035), a frequency counter (Bruker ER041XK), and a variable temperature controlcontinuous flow N_2 cryostat (Bruker B-VT 2000). The g-factor corrections were performed using 2,2-diphenyl-1-picrylhydrazyl (g = 2.0037) as a standard. EPR spectra were simulated with WINEPR SimFonia software.

Detail Synthetic Procedures

Synthesis of 2,3-Dimethyl-2,3-bis(hydroxylamino)butane (BHA)

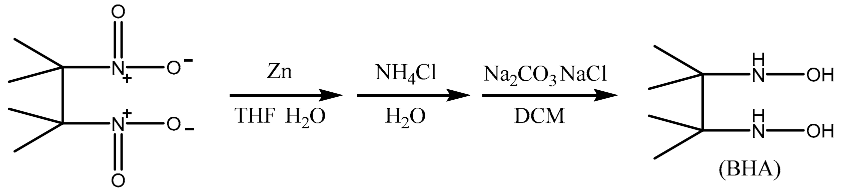

The procedure was followed a similar method that previously published.[1] 11.7 g 2,3-dimethyl-2,3-dinitrobutane and 18.0 g of Zn (dust) in 200 mL THF and 40 mL H_2O was filled into a 1000 mL three-neck flask, and cooled down around 0 ºC in an ice bath under argon. Then the solution of 28.7 g NH_4Cl in 150 mL distilled H_2O was added slowly dropwise (2 hours) under vigorous (mechanical) stirring, meanwhile keep the

temperature of whole system not more than 5 °C. After addition was completed, the reaction was kept stirring for another 1.5 h at the same temperature, and an additional 0.5 h at room temperature. Then the white-gray precipitate of Zn slurry was filtered off, and was washed with THF (30 mL ×3). Filtrate was evaporated to viscous residue, cooled to -15°C (the mixture was kept in the freezer for 2 h). After that the flask was filled with argon and a mixture of 35 g Na_2CO_3 and 20 g NaCl salts was added at once. The flask was vigorously shaken for 15 min to guarantee homogeneous of whole mixture, and after homogenization the white solid was charged into a Soxhlet apparatus, protected under argon atmosphere, then refluxed and extracted with 300 mL of dichloromethane (72-96 h). There is white precipitate gradually appearing in DCM in the bottom flask, and the white precipitate was filtered off by Büchner funnel, washed with dichloromethane (3×30 mL), hexane (3×30 mL) and dried on air. Yield = 3.5 g (30%). **M.p.** 160 -161°C. **FT-IR** (powder, ν/cm^{-1}): 3257 (vs and broad, ν_{OH}), 2987 (vs, $\nu_{C\text{-}H}$), 1479-1374 (vs, several bands), 1261 (s), 1178 (vs), 1145 (vs), 1080 (s), 1035 (vs), 989 (m), 952 (vs), 904 (vs), 852 (m), 790 (m), 690 (m).

Synthesis of 2,7-dibromo-10H-phenothiazine (1)

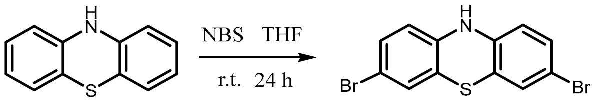

(1)

10H-phenothiazine (4.0 g, 20 mmol) was dissolved in THF (20 mL) at 0 °C and the resulting yellow solution was treated dropwise with a solution of N-bromosuccinimide (NBS, 7.5 g, 42 mmol) in THF (100 mL) under vigorous stirring over a period of 1 h. The resulting dark green solution was allowed to warm to r.t. and stirred for a further 24 h. Two thirds of the solvent were then removed under reduced pressure and the mixture was rereduced by adding a solution of ascorbic acid (1.37 g, 10 mmol) in ethanol/water (1:1, 20 mL). The resulting clear yellow to light orange solution was treated with water (200 mL) to afford the crude product as an amorphous precipitate, which was filtered over a Buechner funnel and recrystallized from acetone/DCM (1:1, 100 mL), giving the product as light green crystals after some days of slow

crystallization. The product was filtered, washed with cold acetone, and dried in vacuo. The combined yield was 2.5 g, 35%. **^{1}H NMR** (DMSO-*d*6, 250.0 MHz), δ ppm: 8.87 δ (s, 1H), 7.16 δ (d, 2H), 7.14 δ (s, 2H), 6.62 δ (d, 2H). **MS** : m/z 357.4 (M+).

Synthesis of 2,7-dithiophen-2-yl-10H-phenothiazine (2)

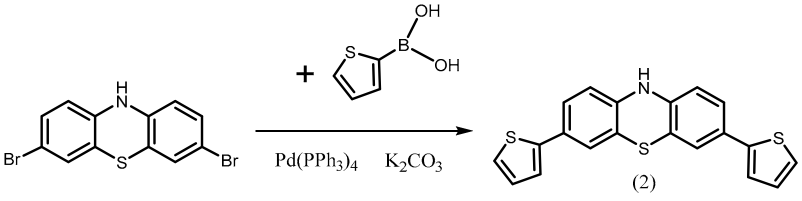

2,7-dibromo-10H-phenothiazine (500 mg, 1.4 mmol) was dissolved in a mixture of THF and water (10:1, 25 mL). To the resulting green solution, a finely ground mixture of thiophene-2-boronic acid (500 mg, 3.9 mmol), K_2CO_3 (1.0 mg, 7.2 mmol) and $Pd(PPh_3)_4$ (40 mg, 2%) was added immediately under vigorous stirring. Argon bobbling for 20 mins. Then the reaction mixture was kept under reflux conditions at for 5 h and was allowed to cool to room temperature. The product precipitated during cooling. The suspension was diluted with deionized water (100 mL) to increase precipitation. The crude product was filtered over a Buechner funnel, washed with hexane and air dried. 300 mg, 60% product acquired. **MS** : m/z 363.2 (M+).

Synthesis of 2,7-di(thiophen-2-yl)-10-(benzaldehyde-4-yl)phenothiazine (3)

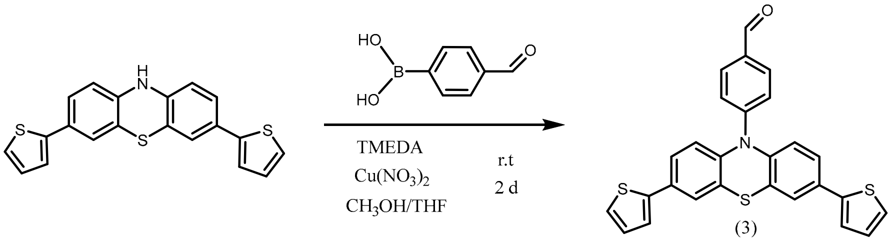

2,7-dithiophen-10H-phenothiazine (200 mg, 0.55 mmol), TMEDA (14.9 mg, 5%) and $Cu(NO_3)_2$ (31.2 mg, 10%) were dissolved in mixture of THF and methanol (10:1, 20 mL). Then 4-formylphenylboronic acid (165 mg, 1.1 mmol) which was dissolved in methanol was added to the flask dropwise. Air was used to bubble the solution. Keep the reation at r.t. for 48 h. The reaction mixture was diluted with water (100 mL), extracted with dichloromethane for several times, washed with brine (100 mL), dried over anhydrous $MgSO_4$, filtered and concentrated under reduced pressure and then was

purified by column chromatography (hexane / toluene = 1 : 1) (yield, 206 mg, 80%). The product **3** is yellow crystal. **^{1}H NMR** (CD_2Cl_2, 250.0 MHz), δ ppm: 9.99 δ (s, 1H), 7.90-7.95 δ (d, 2H), 7.61-7.64 δ (d, 2H), 7.44-7.49 δ (d, 2H), 7.41-7.42 δ (s, 2H), 7.35-7.38 δ (d, 2H), 7.32-7.35 δ (d, 2H), 7.11-7.16 δ (t, 2H), 7.00-7.04 δ (d, 2H). Calculated MW = 467.1 ; **FD. Mass**: 467.7.

Synthesis of 2,7-di(thiophen-2-yl)-10-(benzimidazolidine-4-yl)phenothiazine (4)

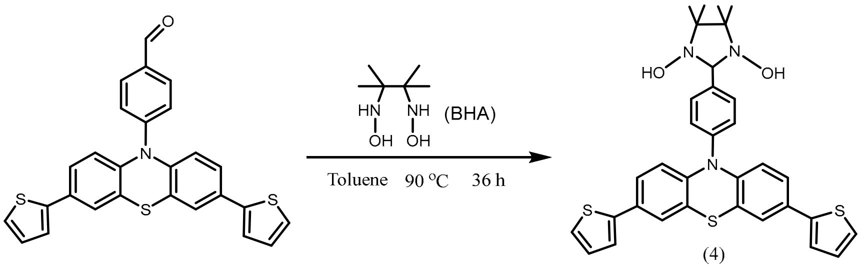

Precursor 3 (50 mg, 0.1 mmol) and 3 equiv 2,3-dimethyl-2,3bis(hydroxylamino)butane (BHA, 45 mg) were charged into a flask, evacuated and kept under argon. Toluene (10 mL) was added to the flask from syringes and was kept argon bubbling for 20 mins. Then the system was heated to 90°C for 36 h. The color of mixture turned to orange. The mixture was washed by MeOH for two times. Then the solvent was evaporated and the orange solid does not need further purification for synthesis of next step.

Synthesis of 2,7-di(thiophen-2-yl)-10-(1-oxyl-3-oxy-4,4,5,5-tetramethylimidazo-line-4-yl)phenothiazine (5, PDTN-NN) and 2,7-di(thiophen-2yl)-10-(1-oxyl-4,4,5,5-tetramethylimidazoline-4-yl)phenothiazine (6, PDTN-IN)

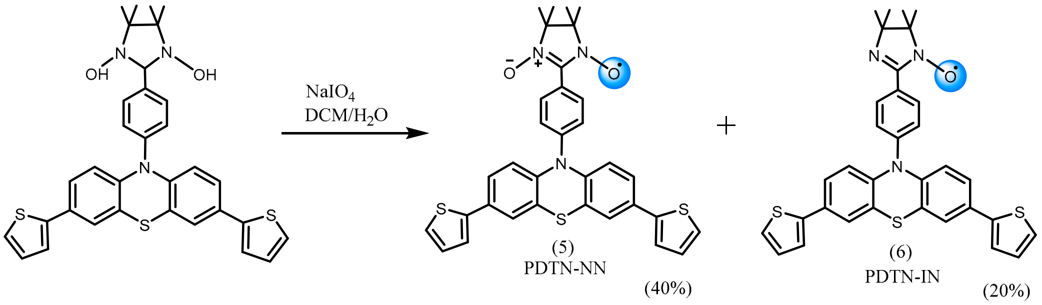

Compound **4** was dissolved in 15 mL DCM and charged into a flask. Then the solution was slowly added with 1.1 equiv $NaIO_4$ dissolved in mixture of 10 mL distilled water and 10 mL DCM. The reaction was kept in ice bath for about 1 hour. **5** and **6** were acquired simultaneously as mixture products. When the color of mixture turned from

orange to dark green, the proceeding of oxidation could be stopped. **5** has a smaller rf value and moved slower than **6** on TLC board. In addition the apparent color of **5** which is green, and **6** which is orange, also have a big difference that could easily distinguished on the TLC board. The mixture products were separated by column chromatography providing a MS-FD MW= 594.1 g/mol PDTN-NN, the yield is 55%. And over oxidized product PDTN-IN could also isolated by column chromatography providing a mass of **MS-FD** MW= 577.9 g/mol, the yield is 20%. **UV-Vis** (measured in DCM) results: **5**, λ_{max} = 640 nm (specific UV-Vis absorption of NN group from 550 nm to 700 nm). **6**, λ_{max} = 480 nm (specific UV-Vis absorption of IN group from 470 nm to 520 nm). **EPR** spectrum (measured in DCM) present the radical **5** g factor = 2.00709 and hyperfine coupling a_N = 7.67 G, and the radical **6** g factor = 2.00644 and hyperfine coupling a_{N1} = 4.51 G, a_{N2} = 9.02 G.

The formation of diradical cations (7, PTD-NN) and (8, PTD-IN)

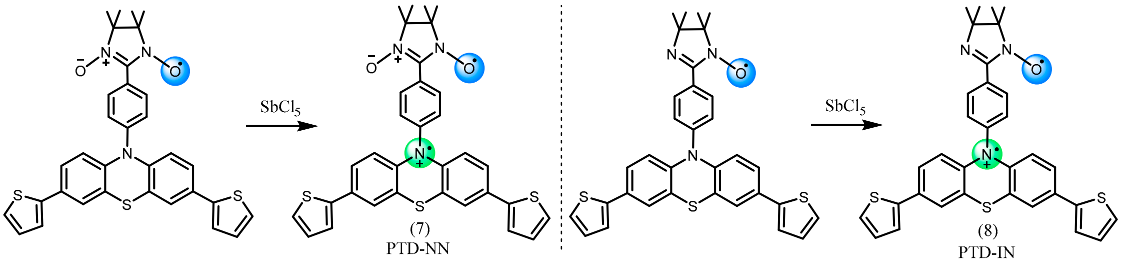

The last step of formation of diradical cations **PTD-NN 7** and **PTD-IN 8** were performed by adding oxidant $SbCl_5$ (10^{-3} M in DCM) to the solution of **5** (10^{-4} M in DCM) and **6** (10^{-4} M in DCM), respectively by microinjector. The solution was stirred at room temperature for 30 minutes, and the color of the solution turned deep pink. The highest conversions of diradical cations in the reactions are approximate at the ratio 2:1 of oxidant $SbCl_5$ to **5** and **6**. The whole reaction processes were monitored by **UV-Vis** absorption measurement and **EPR** measurements.

Synthesis of 10-(benzaldehyde-4-yl)phenoxazine (9)

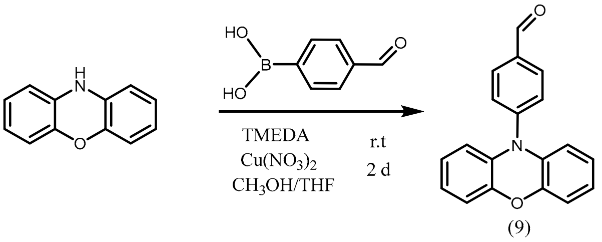

10H-phenoxazine (180 mg, 0.98 mmol), TMEDA (14.9 mg, 5%) and $Cu(NO_3)_2$ (31.2 mg, 10%) were dissolved in mixture of THF and methanol (10:1, 20 mL). Then 4-formylphenylboronic acid (165 mg, 1.1 mmol) which was dissolved in methanol was added to mixture solution. Air was used to bubble the solution. Keep the reation at r.t. for 48 h. The reaction mixture was diluted with water (100 mL), extracted with dichloromethane for several times, washed with brine (100 mL), dried over anhydrous $MgSO_4$, filtered, and concentrated under reduced pressure and then was purified by column chromatography (hexane / toluene = 1 : 1) (yield, 240 mg, 85%). The product is yellow crystal. **^{1}H NMR** (CD_2Cl_2, 250.0 MHz), δ ppm: 9.98 δ (s, 1H), 7.91-7.93 δ (d, 2H), 7.46-7.51 δ (d, 2H), 6.68-6.72 δ (d, 2H), 6.55-6.65 δ (m, 4H), 5.95-5.98 δ (d, 2H). Calculated MW = 287.3 ; **FD. Mass**: 287.7.

Synthesis of 10-(benzimidazolidine-4-yl)phenoxazine (10)

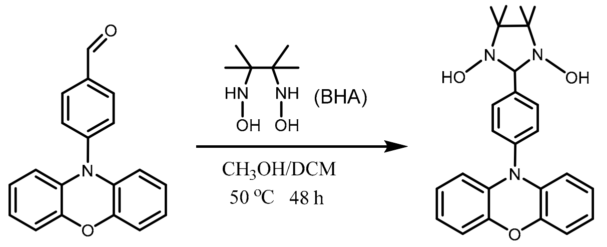

(10)

Precursor **9** (50 mg, 0.17 mmol) and 2 equiv 2,3-dimethyl-2,3-bis(hydroxyl-amino)butane (BHA, 50 mg) were charged into a flask, evacuated and kept under argon. Toluene (10 mL) was added to the flask from syringes and was kept argon bubbling for 20 mins. Then the system was heated to 90°C for 36 h. The color of mixture turned to orange. The mixture was washed by MeOH for several times. The solvent was evaporated and the orange solid does not need further purification for synthesis of next step.

Synthesis of 10-(1-oxyl-3-oxy-4,4,5,5-tetramethylimidazoline-4-yl)phenoxazine (11, PO-NN) and 10-(1-oxyl-4,4,5,5-tetramethylimidazoline-4-yl)phenoxazine (12, PO-IN)

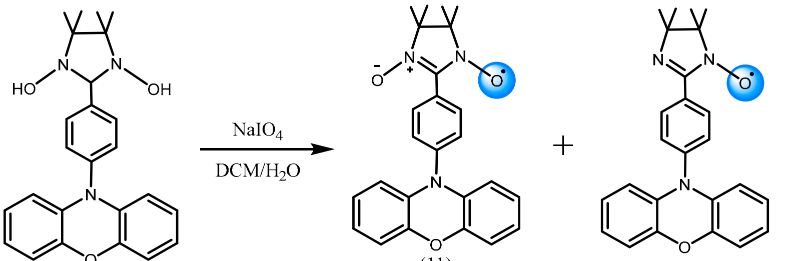

(11) PO-NN (45%)   (12) PO-IN (20%)

Compound **10** was dissolved in 15 mL DCM and charged into a flask. Then the solution was slowly added with 1.1 equiv $NaIO_4$ dissolved in mixture of 10 mL distilled water and 10 mL DCM. The reaction was kept in ice bath for about 1 hour. **11** and **12** were acquired simultaneously as mixture products. When the color of mixture turned from orange to dark green, the proceeding of oxidation could be stopped. **11** has a smaller rf value and moved slower than **12** on TLC board. In addition the apparent color of **11** which is light blue, and **12** which is orange, also have a big difference that could easily distinguished on the TLC board. The mixture products were separated by column chromatography providing a MS-FD MW= 414.5 g/mol PDTN-NN, the yield is 45%. And over oxidized product PDTN-IN could also isolated by column chromatography providing a mass of **MS-FD** MW= 398.5 g/mol, the yield is 20%. **UV-Vis** (measured in DCM) results: **11,** λ_{max} = 617 nm (specific UV-Vis absorption of NN group from 550 nm to 700 nm). **12,** λ_{max} = 480 nm (specific UV-Vis absorption of IN group from 470 nm to 520 nm). **EPR** spectrum (measured in DCM) present the radical **11** g factor = 2.00707 and hyperfine coupling a_N = 7.72 G, and the radical **12** g factor = 2.00634 and hyperfine coupling a_{N1} = 4.25 G, a_{N2} = 8.51 G.

The formation of diradical cations (13, POD-NN) and (14, POD-IN)

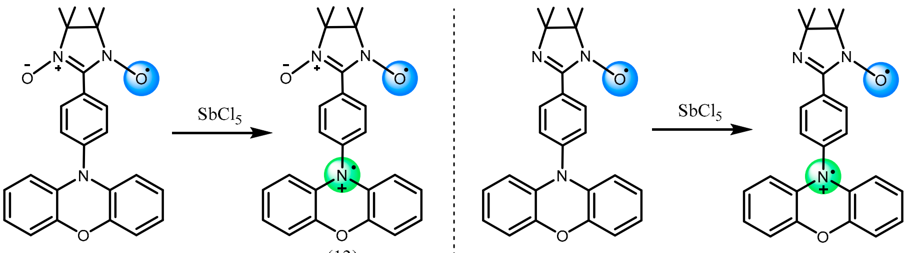

(13) POD-NN   (14) POD-IN

The last step of formation of diradical cations **POD-NN 13** and **POD-IN 14** were

performed by adding oxidant $SbCl_5$ (10^{-3} M in DCM) to the solution of **11** (10^{-4} M in DCM) and **12** (10^{-4} M in DCM), respectively by microinjector. The solution was stirred at room temperature for 30 minutes, and the color of the solution turned deep pink. The highest conversions of diradical cations in the reactions are approximate at the ratio 2:1 of oxidant $SbCl_5$ to **11** and **12**. The whole reaction processes were monitored by **UV-Vis** absorption measurement and **EPR** measurements.

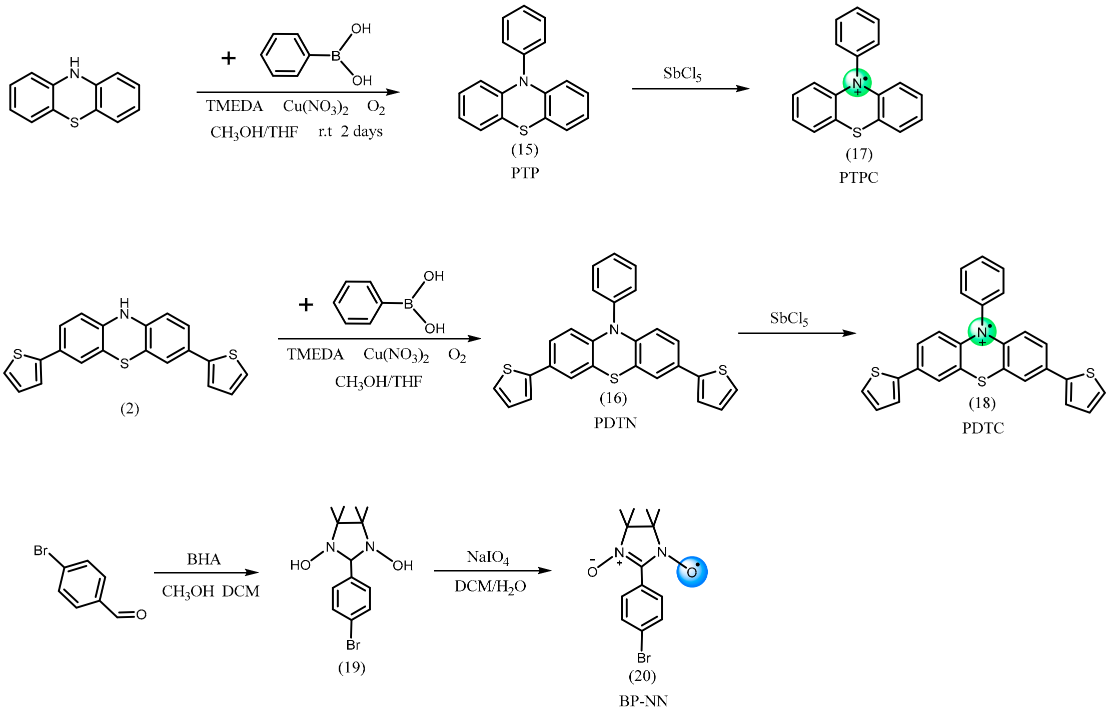

Scheme S1. Synthesis of cation monoradicals PTPC, PDTC and BP-NN.

Synthesis of 10-phenyl-10*H*-phenothiazine (15)

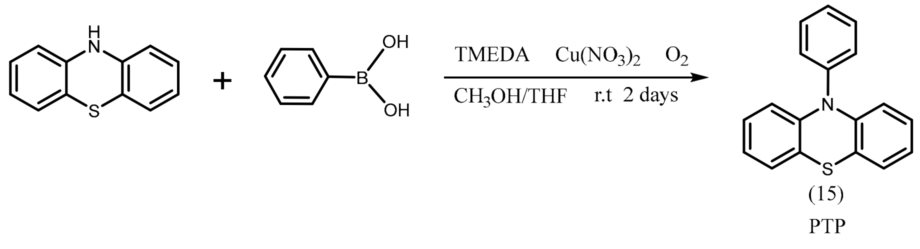

10H-phenothiazine (200 mg, 1.01 mmol), TMEDA (11.6 mg, 5%) and $Cu(NO_3)_2$ (24.2 mg, 10%) were dissolved in mixture of THF and methanol (10:1, 20 mL). Then phenylboronic acid (122 mg, 1.1 mmol) which was dissolved in methanol was added

to the flask dropwise. Air was used to bubble the solution. Keep the reation at r.t. for 24 h. The reaction mixture was diluted with water (100 mL), extracted with dichloromethane for several times, washed with brine (100 mL), dried over anhydrous $MgSO_4$, filtered, and concentrated under reduced pressure and then was purified by column chromatography (hexane / toluene = 1 : 1). **^{1}H NMR** (CD_2Cl_2, 250.0 MHz), δ ppm: 7.58-7.62 δ (t, 2H), 7.50 δ (t, 1H), 7.38-7.43 δ (d, 2H), 7.00-7.05 δ (d, 2H), 6.82-6.88 δ (m, 4H), 6.18-6.22 δ (d, 2H). (yield, 151 mg, 55%). The product is light yellow crystal. **MS** : m/z 275 (M+).

Synthesis of 2,7-di(thiophen-2-yl)-10-(phenyl-4-yl)phenothiazine (16, PDTN)

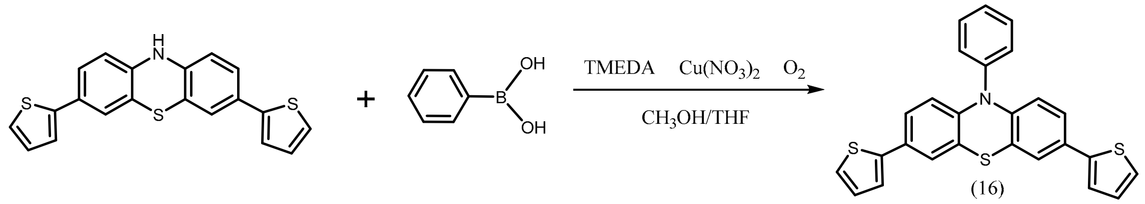

2,7-dithiophene-10H-phenothiazine **2** (150 mg, 0.41 mmol), TMEDA (11.6 mg, 8%) and $Cu(NO_3)_2$ (15.2 mg, 10%) were dissolved in mixture of THF and methanol (10:1, 20 mL). Then phenylboronic acid (60 mg, 0.55 mmol) which was dissolved in methanol was added to the flask dropwise. Air was used to bubble the solution. Keep the reation at r.t. for 24 h. The reaction mixture was diluted with water (100 mL), extracted with dichloromethane for several times, washed with brine (100 mL), dried over anhydrous $MgSO_4$, filtered and concentrated under reduced pressure and then was purified by column chromatography (hexane / toluene = 1 : 1). **^{1}H NMR** (CD_2Cl_2, 250.0 MHz), δ ppm: 7.55-7.60 δ (t, 2H), 7.52 δ (t, 1H), 7.48-7.51 δ (d, 2H), 7.31-7.41 δ (m, 6H), 7.15-7.05 δ (m, 4H), 6.18-6.22 δ (d, 2H). (yield, 100 mg, 56%). The product is light yellow crystal. **MS** : m/z 440.7 (M+).

The formation of cation radicals (17, PTPC) and (18, PDTC)

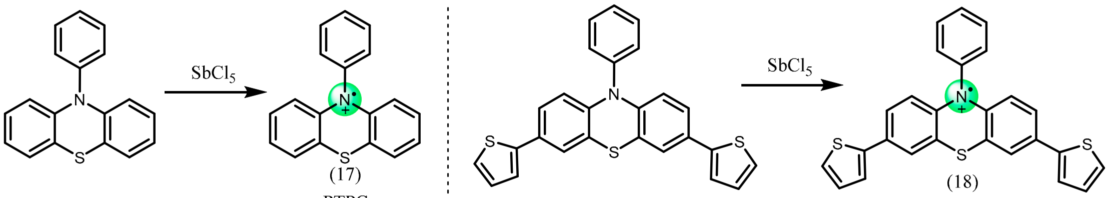

The last step of formation of cation radicals **PTPC 17** and **PDTC 18** were performed by adding oxidant $SbCl_5$ (10^{-3} M in DCM) to the solution of **15** (10^{-4} M in DCM) and **16** (10^{-4} M in DCM), respectively by microinjector. The solution was stirred at room temperature for 30 minutes, and the color of the solution turned red. The final ratio of oxidant $SbCl_5$ to **15** and **16** is 5:1. The whole reaction processes were monitored by EPR measurements. **EPR** spectrum (measured in DCM) present the radical **17** g factor = 2.0052 and hyperfine coupling a_N = 6.52 G, and the radical **18** g factor = 2.0052 and hyperfine coupling a_N = 6.55 G.

NMR spectra

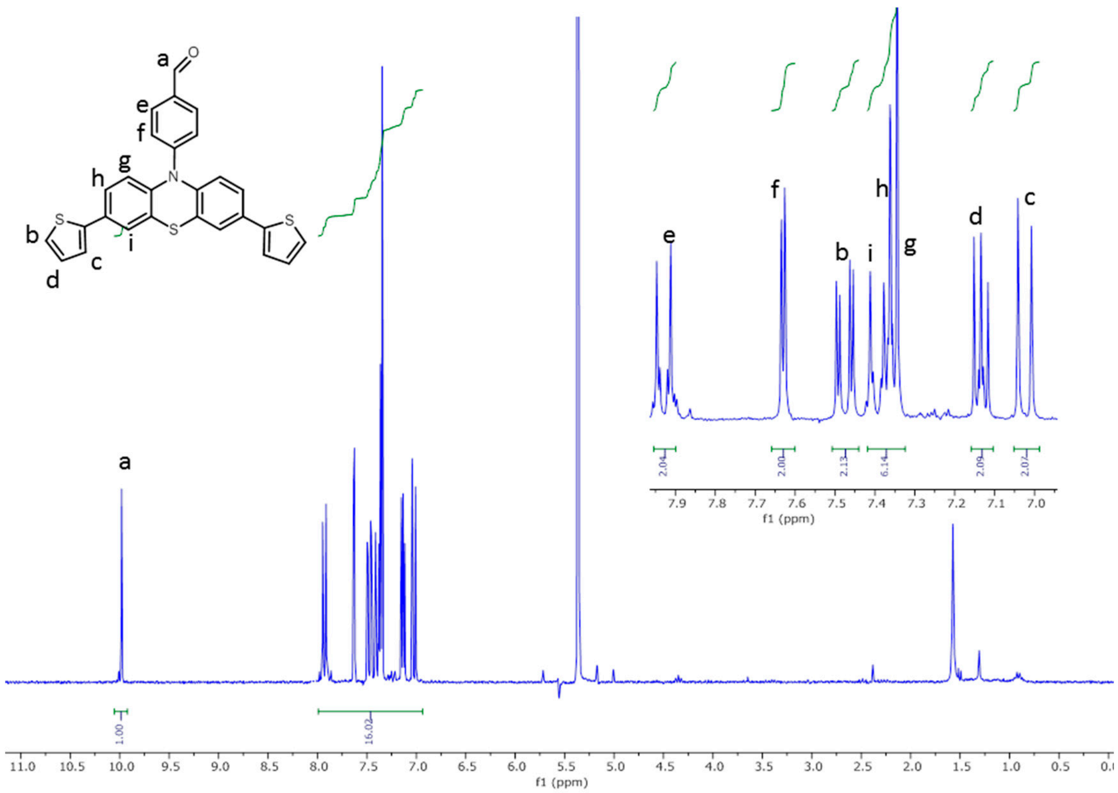

Figure S1. ^{1}H NMR spectrum (250.0 MHz) of **3** in CD_2Cl_2 at r.t.

Supporting UV-Vis Absorption Spectra

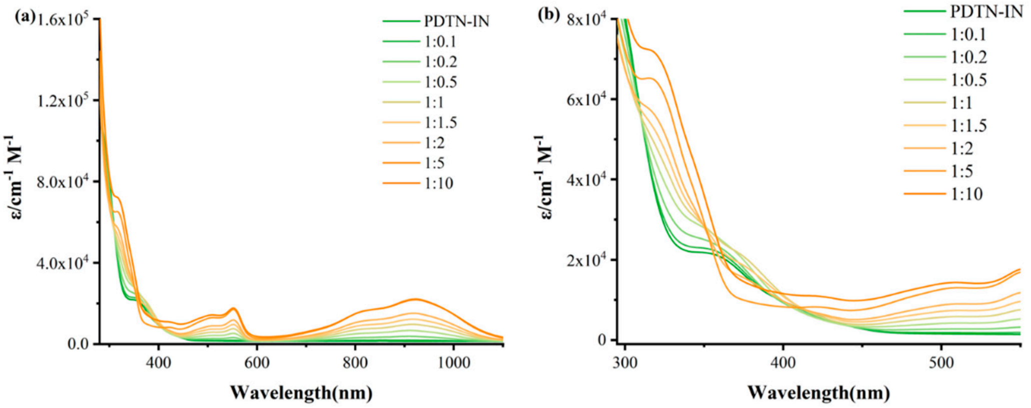

Figure S2. (a) UV-Vis absorption spectra of the whole oxidation titration process of PDTN-IN by adding different ratios of oxidants $SbCl_5$ recorded in DCM (~10^{-4} M) at r.t. (b) Amplification of optical absorption spectra in the range from 290 nm to 550 nm.

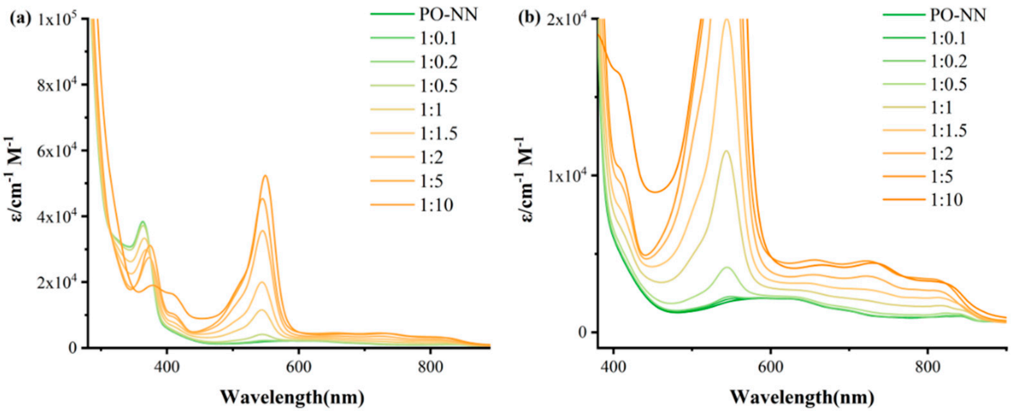

Figure S3. (a) UV-Vis absorption spectra of the whole oxidation titration process of PO-NN by adding different ratios of oxidants $SbCl_5$ recorded in DCM (~10^{-4} M) at r.t. (b) Amplification of optical absorption spectra in the range from 380 nm to 900 nm.

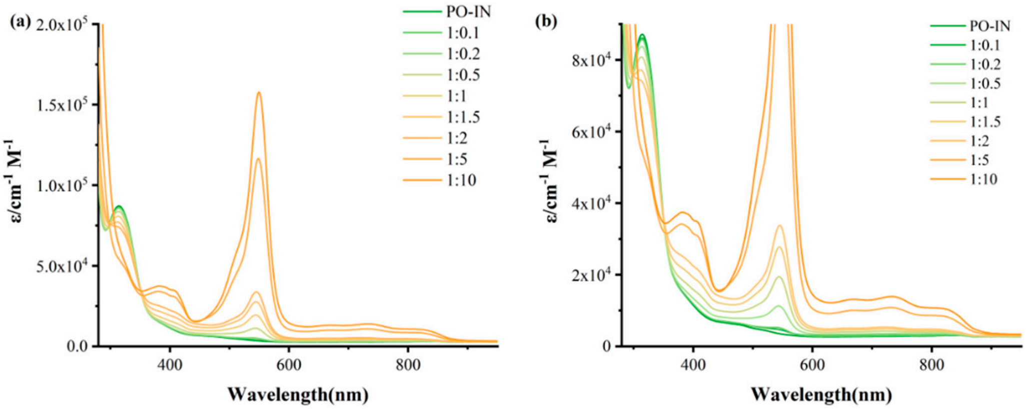

Figure S4. (a) UV-Vis absorption spectra of the whole oxidation titration process of PO-IN by adding different ratios of oxidants $SbCl_5$ recorded in DCM (~10^{-4} M) at r.t. (b) Amplification of optical absorptionspectra in the range from 280 nm to 950 nm.

Supporting Cyclic Voltammetry Measurements

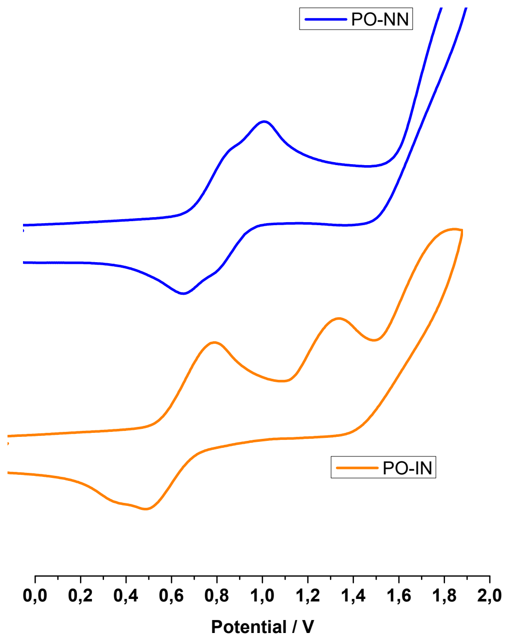

Figure S5. The cyclic voltammetry curves (the oxidation part) of PO-NN, PO-IN recorded in DCM at r.t, respectively. (Bu_4NPF_6, 0.1 M, the scan rate is 100 mV/s)

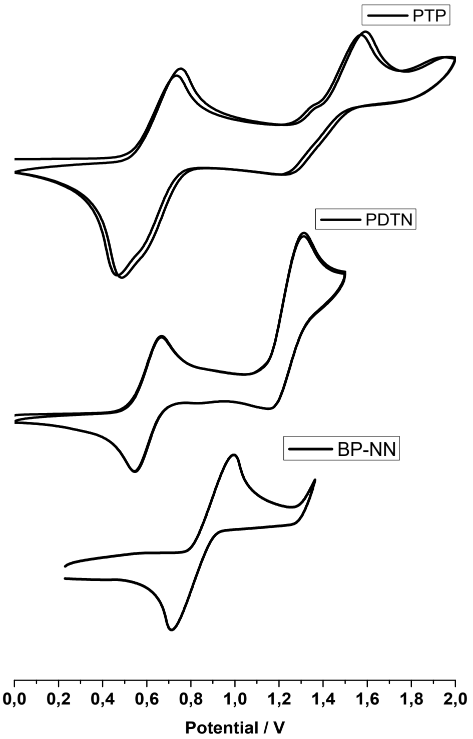

Figure S6. The cyclic voltammetry curves (the oxidation part) of PTP, PDTN, and BP-NN recorded

in DCM at r.t, respectively. (Bu_4NPF_6, 0.1 M, the scan rate is 100 mV/s)

The first oxidation potential is attributed to the first electron losses from tertiary amino of phenothiazine which became cation radical. The other oxidation potential is attributed to the second electron from the same nitrogen of phenothiazine which then became dication. The values of E_{Oxi-1} and E_{Oxi-2} for PTP are higher than those for PDTN, indicating that the electron-donating ability of the thiophene group in PDTN makes the structure easier to oxidize.

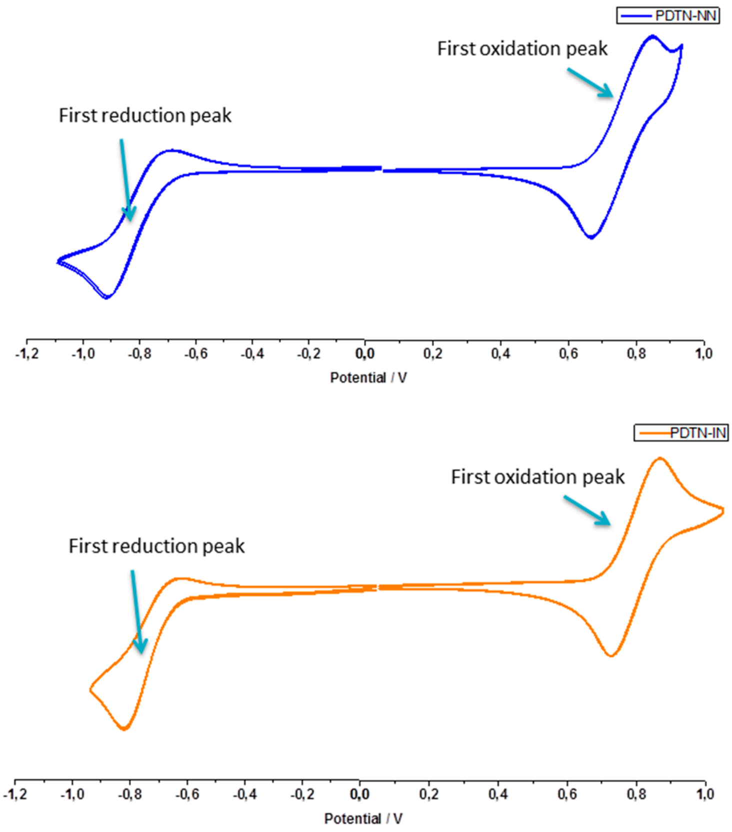

Figure S7. The first oxidation and reduction of cyclic voltammetry curves of PDTN-NN and PDTN-IN recorded in DCM at r.t, respectively. (Bu_4NPF_6, 0.1 M, the scan rate is 100 mV/s)

We examined the cyclic voltammetry (CV) of PDTN-NN and PDTN-IN over a full cycle of oxidation and reduction. The first oxidation potential peak and first reduction potential peak were carried out from Figure S7 for calculating

SOMO, LUMO energy levels and energy gap between them by following the equations ***(a)*** and ***(b)*** listed in Table S1.

$$E_{SOMO} = -(E_{Oxi\text{-}1} - E^{(1/2)}_{Fc/Fc} + 4.8) \text{ eV} \quad \textbf{(a)}$$

$$E_{LUMO} = -(E_{Red\text{-}1} - E^{(1/2)}_{Fc/Fc} + 4.8) \text{ eV} \quad \textbf{(b)}$$

Table S1. The optical and electrochemical energy levels of PDTN-NN and PDTN-IN

	E_{Oxi}, V	E_{Red}, V	E_{SOMO}, eV	E_{LUMO}, eV	ΔE_g, eV	E_{opt}, eV
**PDTN-NN**	0.78	-0.80	-5.12	-3.54	1.58	1.61
**PDTN-IN**	0.80	-0.75	-5.14	-3.59	1.55	2.14

EPR spectra

We simulated the EPR spectra of starting compound of PDTN-NN and its over oxidized state (g = 2.0052, a_N = 6.74G), as shown in Figure S8a and S8b. To analyze the intermediate diradical cation species, we selected an EPR spectrum of the mixed state at an oxidant-to-starting compound ratio of 2:1, which exhibited a nine-line pattern signal. We then combined the EPR spectra of the two pure states (starting compound and over-oxidized state) in a 1:1 ratio, resulting in a new spectrum shown in Figure S8c. It completely doesn't fit the experimental spectrum of mixted state, no matter the number of peak splitting and the position (gauss value) of the signal. This discrepancy indicates the presence of a new signal pattern corresponding to an intermediate radical species formed during the oxidation process. We simulated a diradical species (PTD-NN) with g =2.0063 and septet splitting peaks with (a_{N1} = 3.76G, a_{N2} = 3.25G), as shown in Figure S8d. This simulated spectrum would be checked whether it fits the experimental spectrum of intermediate radical species PTD-NN we expected.

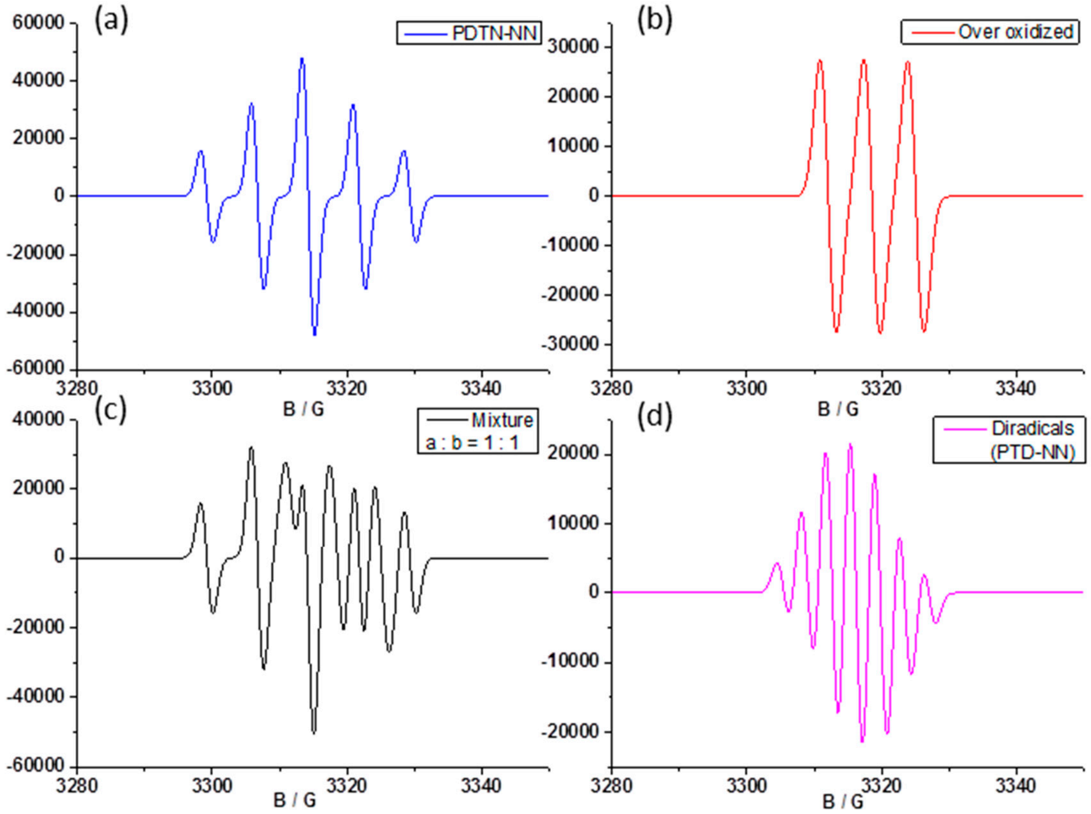

Figure S8. The simulated EPR spectra of (a) PDTN-NN, (b) the over-oxidized state, (c) the mixture of (a) and (b) with the ratio 1 : 1, (d) PTD-NN.

The spectrum shows a clear mono-NN with five lines patterns signals (g = 2.0070) on the left top of Figure S9. There are no difference of EPR spectra between the pure BP-NN sample and the oxidation sample with the $SbCl_5$ ratio 1 : 1 and 1 : 2. That means the NN group is stable at least in the oxidation condition of 2 eq $SbCl_5$. But contrast sample mono-NN group was nearly all decomposed at the oxidation condition of 4 eq $SbCl_5$. The signal peak nearly disappeared, indicating that the unpaired electron in NN group has been eliminated.

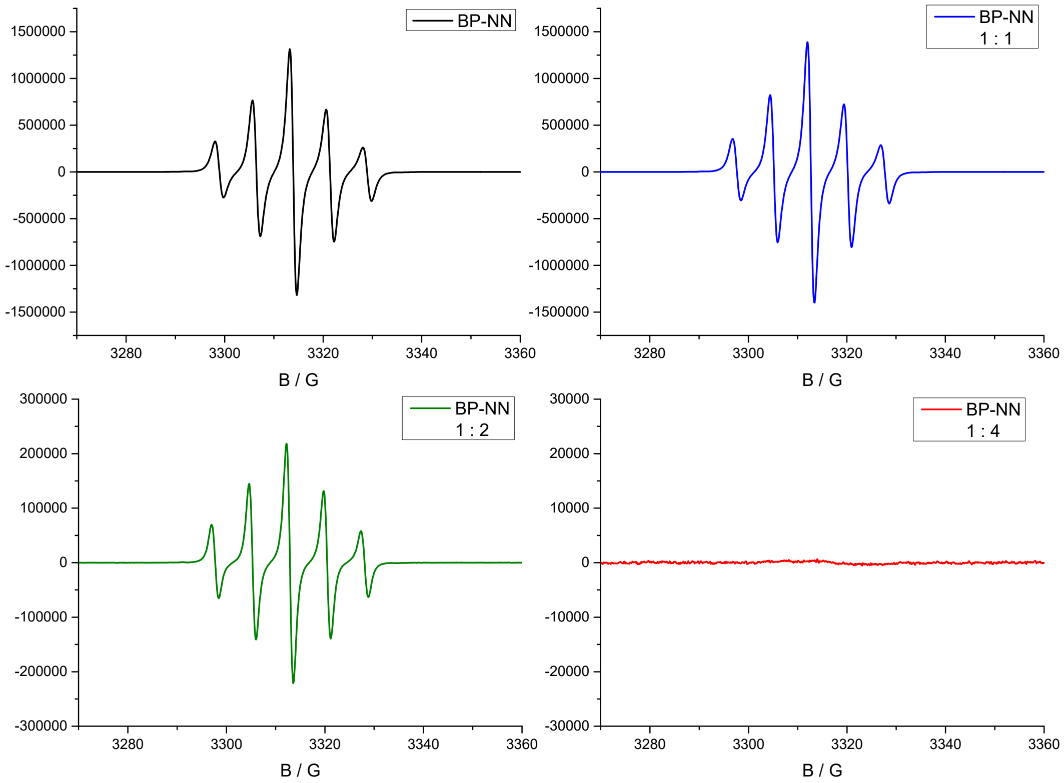

Figure S9. X-band EPR spectra of BP-NN with different ratios of oxidant $SbCl_5$ in DCM (c~10^{-4} M) at r.t.

The three-line pattern signal suggests the presence of a spin-bearing nitrogen, indicating the formation of cation monoradicals (PTPC and PDTC with g = 2.0052 and a_N = 6.5G) in both cases during the oxidation process.

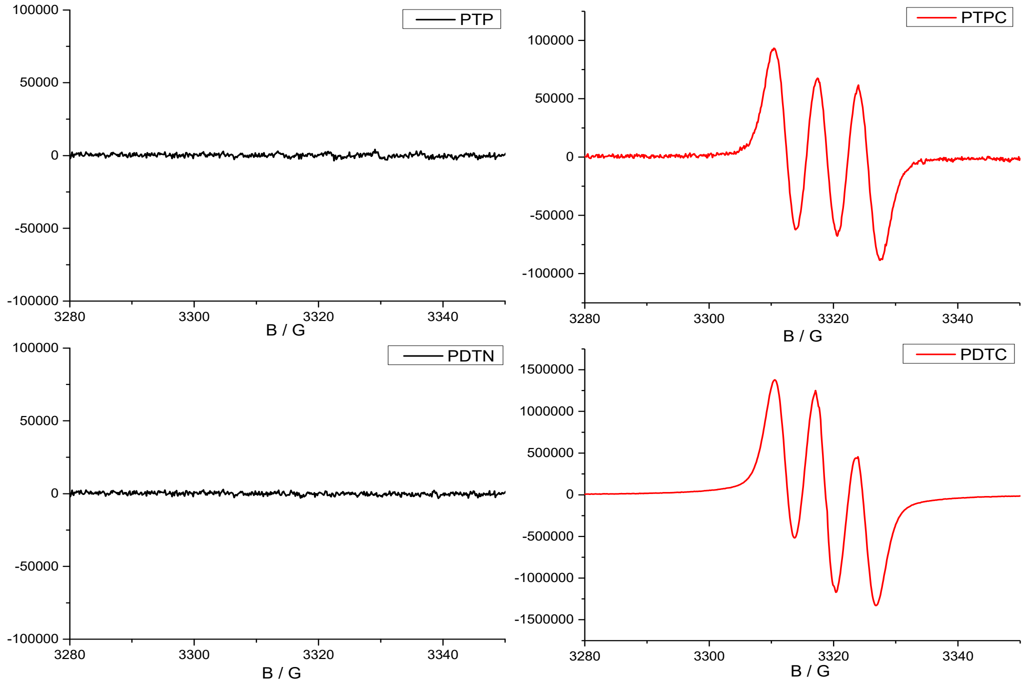

Figure S10. X-band EPR spectra of PTP, PDTN and the oxidized cation monoradicals PTPC and PDTC with oxidant $SbCl_5$ in DCM (c~10^{-4} M) at r.t.

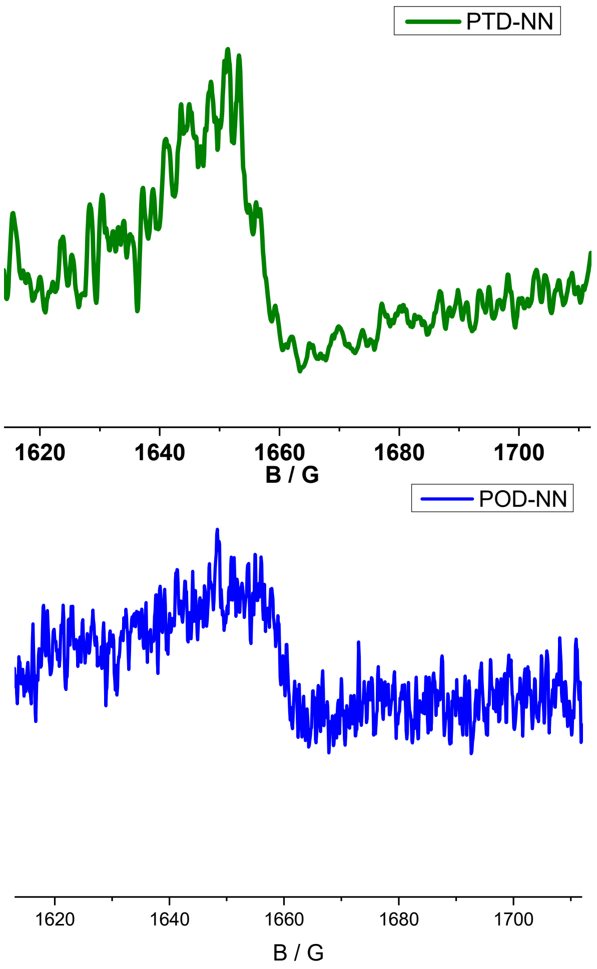

Figure S11. the peak signal appears both in PTD-NN sample (upper) and POD-NN sample (lower) in the forbidden transition ($\Delta_{ms} = 2$) around g ≈ 4 region, measured at 120 K in DCM.

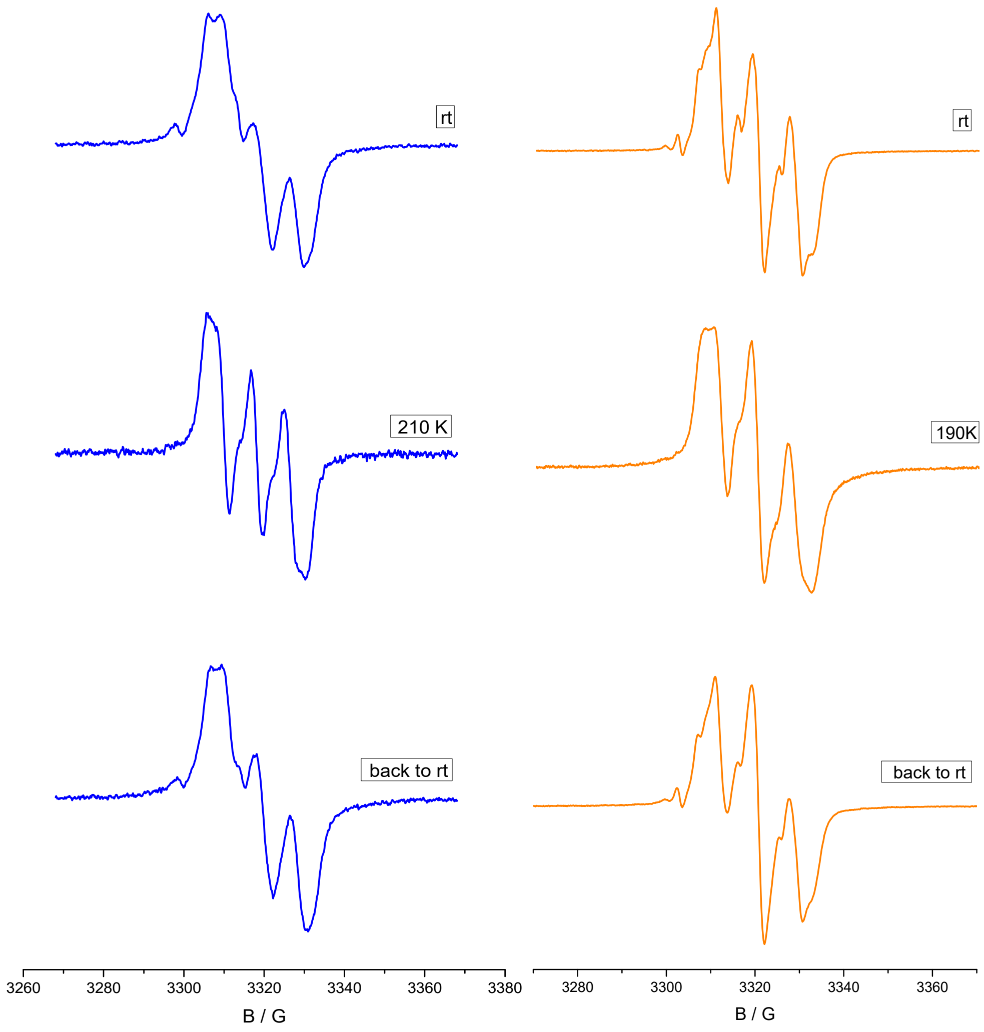

Figure S12. EPR Spectra of intermediate states of PO-NN and PO-IN with temperature dependent reversible features in DCM.

DFT Calculations

Table S2. Summary of diradical cations calculation results

**Diradical cations**	**E_{BS} (Hartree)**	**E_{T} (Hartree)**	**$<S^2>_{BS}$**	**$<S^2>_{T}$**
**PTD-NN**	-2625.463149	-2625.4684608	0.937689	2.020318
**POD-NN**	-1199.0315485	-1199.0330987	0.855647	2.020864
